# Immunometabolic Dysregulation in Obesity: Integrating Macrophage Polarization, Metabolic Reprogramming, and Gut Microbiota Crosstalk

**DOI:** 10.1002/iid3.70470

**Published:** 2026-08-17

**Authors:** Yuanyuan Li, Jingwei Mao, Yiwen Deng, Xiaobo Ge, Limin Nie, Qun Wang, Ruiqing Wang, Jiarun Zhang, Tianli Lyu, Luopeng Zhao, Lu Liu, Bin Li

**Affiliations:** ^1^ Beijing Key Laboratory of Acupuncture Neuromodulation, Department of Acupuncture and Moxibustion, Beijing Hospital of Traditional Chinese Medicine Capital Medical University Beijing China; ^2^ Beijing Key Laboratory of Digital Intelligent Acupuncture Beijing China; ^3^ Beijing University of Chinese Medicine Beijing China; ^4^ The First Affiliated Hospital of Henan University of Chinese Medicine Zhengzhou China

**Keywords:** gut microbiota, inflammation, macrophage, metabolism, obesity

## Abstract

**Background:**

Obesity is characterized by chronic low‐grade systemic inflammation, in which adipose tissue macrophages (ATMs) play a pivotal role in driving immune and metabolic dysfunction.

**Objective:**

This review summarizes current evidence on the roles of ATMs in obese adipose tissue and examines how macrophage polarization and metabolic reprogramming are linked to fatty acid metabolism, insulin resistance (IR), and gut microbiota dysbiosis.

**Recent Findings:**

Previous studies have often addressed macrophage dysfunction, fatty acid metabolism, IR, and gut microbiota alterations as separate processes. In contrast, this review integrates these obesity‐related events within a unified immunometabolic framework centered on ATMs. Obesity induces marked phenotypic shifts and metabolic reprogramming in ATMs, thereby promoting chronic inflammation and IR. Disturbed fatty acid metabolism and gut microbiota dysbiosis further impair metabolic homeostasis and intestinal barrier integrity, reinforcing a vicious cycle of macrophage‐driven immunometabolic dysfunction.

**Conclusions:**

This review highlights the central role of ATM‐mediated immune‐metabolic crosstalk in obesity and emphasizes the therapeutic potential of targeting macrophage dysfunction and gut microbiota alterations to attenuate obesity‐related metabolic complications.

## 1 Introduction

According to *the World Obesity Report 2025*, approximately 1.13 billion adults worldwide are projected to be affected by overweight or obesity by 2030, and obesity has emerged as a critical global public health challenge, contributing to a rising incidence of premature mortality from obesity‐related conditions, thus exacerbating the public health burden. Obesity is characterized by the excessive accumulation of adipose tissue (AT) resulting from an imbalance between energy intake and expenditure. It is strongly associated with a range of serious comorbidities, including metabolic‐associated fatty liver disease, prediabetes, metabolic syndrome, type 2 diabetes mellitus, lipid metabolism disorders, and cardiovascular diseases [1, 2].

AT, comprising white AT (WAT) and brown AT (BAT), plays a central role in the regulation of energy homeostasis. WAT stores excess energy as triglycerides (TAG) during periods of overnutrition and also serves an important endocrine function by secreting various hormones, such as leptin and adiponectin, which help maintain body weight and metabolic balance [3, 4, 5]. In contrast, BAT primarily functions in thermogenesis, converting stored energy into heat via mitochondrial uncoupling respiration. BAT activity is inversely correlated with obesity risk, and cold exposure, along with certain pharmacological agents like β‐adrenergic agonists, has been shown to activate BAT and improve metabolic outcomes [6, 7, 8]. Additionally, recent studies have highlighted the existence of beige fat, a light‐colored AT that can acquire thermogenic properties similar to BAT in response to cold exposure or specific metabolic signals, offering a potential therapeutic target for obesity intervention [9].

AT adapts to excess energy storage through a compensatory expansion characterized by adipocyte proliferation and hypertrophy [10, 11]. Among these, adipocyte proliferation is a relatively benign form of expansion, while hypertrophy is associated with impaired AT function. Adipocyte hypertrophy is characterized by cellular necrosis, increased secretion of pro‐inflammatory mediators, reduced oxygen availability in the local microenvironment (hypoxia), elevated free fatty acid (FFA) efflux, and diminished glucose uptake by adipocytes. These pathological changes contribute to metabolic dysregulation, manifesting as AT fibrosis, ectopic lipid deposition, and insulin resistance (IR) [12, 13, 14, 15, 16]. In addition to adipocytes, pathologically hypertrophic AT contains a substantial number of macrophages and other non‐adipocyte cell types, including various immune cells such as T cells, B lymphocytes, and natural killer cells [17]. Among these, macrophages play a predominant role in both quantity and function [18, 19]. Studies have shown that macrophages are significantly accumulated in the AT of both obese individuals and animal models, with pro‐inflammatory M1 macrophages being the predominant subtype [20, 21, 22].

Many studies have demonstrated that reducing the infiltration of pro‐inflammatory M1 macrophages in AT or promoting their polarization toward the anti‐inflammatory M2 phenotype can effectively reduce body weight and ameliorate metabolic disorders, including impaired glucose tolerance and IR [23, 24, 25]. The macrophage phenotype transformation, as a pivotal mechanism to alleviate obesity‐associated chronic low‐grade inflammation and metabolic dysregulation, has emerged as a promising therapeutic approach. Recent breakthroughs in single‐cell transcriptomics and related technologies have facilitated the discovery of new macrophage subtypes, including AT‐associated CD9^+^ macrophages, lipid‐associated macrophages (LAM), and norepinephrine‐degrading macrophages (sympathetic neuron‐associated macrophage [SAM]) [26]. These discoveries have significantly advanced our comprehension of the diverse roles of macrophages in the local microenvironment, particularly in the context of obesity and metabolic regulation [27, 28, 29]. Future strategies aimed at precisely modulating specific macrophage subtypes hold great potential for developing targeted therapies to combat obesity and its associated metabolic complications.

The expansion of AT and the damage to adipocytes lead to the recruitment and activation of macrophages, which typically undergo a shift from the anti‐inflammatory M2 phenotype to the pro‐inflammatory M1 phenotype [30]. M1 macrophages exacerbate chronic low‐grade inflammation within AT by secreting inflammatory cytokines such as tumor necrosis factor‐α (TNF‐α), interleukin (IL)‐6, and IL‐1β, thereby disrupting insulin signaling pathways and inducing IR [31]. Furthermore, the activation of adipose tissue macrophages (ATMs)‐mediated inflammation promotes the excessive release of fatty acids, particularly FFAs, which contribute to oxidative stress and further aggravate IR [32]. These fatty acids also facilitate the accumulation of visceral fat, which not only impairs insulin action but also disrupts systemic metabolic balance through the altered release of FFAs and adipokines such as adiponectin and leptin [33]. Importantly, these pathological events are not restricted to AT itself, but are also shaped by inter‐organ communication. Recent evidence suggests that gut microbiota dysbiosis, microbial metabolites, and increased intestinal permeability can influence ATM activation, inflammatory tone, and host metabolic homeostasis, thereby linking the gut and AT in obesity progression.

Previous studies have often examined ATM polarization, fatty acid metabolism, IR, and gut microbiota dysbiosis as relatively independent processes in obesity. However, accumulating evidence indicates that these mechanisms are closely interconnected rather than isolated. In obese AT, nutrient excess, adipocyte hypertrophy, hypoxia, and lipotoxic stress drive the recruitment and phenotypic reprogramming of ATMs, promoting a pro‐inflammatory and metabolically dysregulated state. These dysfunctional ATMs not only amplify local inflammation but also disrupt lipid homeostasis by enhancing lipolysis, FFA release, and ectopic lipid deposition, thereby impairing insulin signaling in AT and peripheral metabolic organs. Meanwhile, gut microbiota dysbiosis and increased intestinal permeability further intensify systemic inflammation and metabolic endotoxemia, which in turn exacerbate ATM activation and AT dysfunction.

Thus, macrophages should be regarded not merely as one component of obesity‐related inflammation, but as a central cellular hub linking immune activation, disordered fatty acid metabolism, IR, and microbiota‐host interactions. On this basis, we selected these mechanisms because they represent major and mechanistically interconnected drivers of obesity‐associated metabolic dysfunction. Accordingly, this review summarizes current knowledge on the origin, heterogeneity, polarization, accumulation, and metabolic reprogramming of ATMs, and discusses how ATM‐centered crosstalk with lipid metabolism, insulin signaling, and gut microbiota contributes to obesity progression and related metabolic complications.

## 2 Literature Search Strategy

This article was designed as a narrative review informed by a structured literature search conducted in PubMed, Web of Science, Scopus, and Embase for studies published up to October 2025. A narrative review approach was chosen because the scope of this article is to integrate heterogeneous evidence spanning mechanistic studies, animal experiments, translational research, and clinical observations, rather than to address a narrowly defined question suitable for quantitative synthesis. In particular, the relationships among ATM polarization, metabolic reprogramming, fatty acid metabolism, IR, and gut microbiota dysbiosis involve multiple interacting biological processes, diverse study designs, and variable outcome measures, making a conventional systematic review or meta‐analysis less appropriate for the present objective. The main search terms included combinations of “obesity,” “adipose tissue macrophages,” “macrophage polarization,” “metabolic reprogramming,” “fatty acid metabolism,” “insulin resistance,” “gut microbiota,” “inflammation,” and “adipose tissue,” using Boolean operators such as AND and OR where appropriate.

We primarily included original research articles and high‐quality review articles published in English that were directly relevant to the role of ATMs in obesity‐associated inflammation and metabolic dysfunction. Particular attention was given to studies addressing fatty acid metabolism, IR, gut microbiota dysbiosis, and macrophage‐related immunometabolic mechanisms. Articles with limited relevance, duplicate content, or insufficient mechanistic information were excluded. Additional relevant studies were identified through manual screening of the reference lists of key publications.

## 3 Origin and Function of ATMs

Macrophages are pivotal components of the innate immune system, and their polarization is intricately linked to immune defense, inflammatory responses, and tissue remodeling [34]. Under normal physiological conditions, macrophages are ubiquitously distributed across tissues and form a self‐sustaining population. These resident macrophages are replenished by monocyte recruitment from the circulation. Monocytes, released from the bone marrow as precursor cells, migrate into tissues and differentiate into specialized macrophages after crossing the endothelium. Based on their origin, macrophages can be categorized into two groups: those derived from yolk sac progenitors during embryogenesis and those derived from bone marrow precursor cells [35, 36]. ATMs primarily originate from resident macrophages and monocyte‐derived recruited macrophages. Unlike most tissue‐resident macrophages, which are derived from yolk sac precursors and contribute to tissue remodeling and homeostasis, ATMs exhibit unique characteristics and functional roles [27].

**Figure 1:**
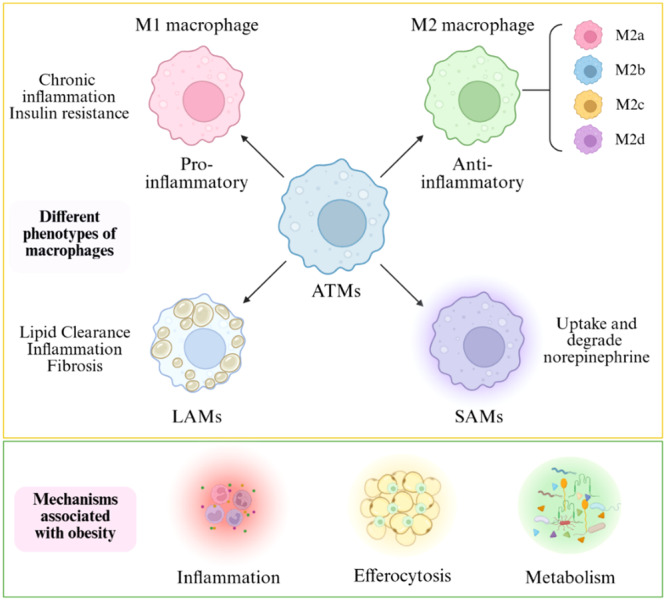
Classification of macrophages and potential mechanisms associated with obesity. ATMs, adipose tissue macrophages; LAMs, lipid‐associated macrophages; SAMs, sympathetic neuron‐associated macrophages.

Since the discovery over a century ago, macrophages have been continuously reclassified based on their phenotypic and functional characteristics. Initial classification identified pro‐inflammatory classically activated macrophages and anti‐inflammatory alternatively activated macrophages, which were later formalized as M1 and M2 macrophages [37, 38, 39]. Further research subdivided M2 macrophages into four subtypes (M2a, M2b, M2c, and M2d) based on their inducing factors, secreted products, and functions [40, 41]. Recent studies identified additional LAM subtypes associated with obesity, including CD9^+^, TREM2^+^, PLIN2^+^, and so on, significantly expanding the known diversity of macrophage phenotypes in AT [42, 43, 44, 45, 46, 47, 48]. Notably, SAMs, a specialized subset, have been found to take up and degrade norepinephrine, thereby suppressing lipolysis and contributing to obesity [49]. These findings underscore the profound impact of microenvironmental changes on macrophage phenotype and function, offering critical insights into the mechanisms underlying obesity, its progression, and related complications (Figure 1).

ATMs represent a highly heterogeneous population of cells with substantial phenotypic plasticity, strongly influenced by the microenvironment [50, 51]. In both genetically and diet‐induced obesity, macrophages constitute the predominant immune cell population in AT. While under normal conditions macrophages account for approximately 10% of AT immune cells, their proportion increases to 40%–50% in obesity [20, 21]. As AT expands, local hypoxia develops due to insufficient vascular oxygen supply, resulting in adipocyte necrosis, enhanced chemokine secretion, and dysregulated fatty acid metabolism, which collectively drive macrophage infiltration [52]. These macrophages form a crown‐like structure (CLS) around necrotic adipocytes through efferocytosis; however, this process is accompanied by the release of pro‐inflammatory mediators such as TNF‐α, IL‐6, and IL‐1β, as well as lipid intermediates, exacerbating inflammation and IR [53, 54]. Furthermore, macrophages regulate fatty acid uptake, storage, and metabolism, but their hyperactivation can lead to excessive fatty acid efflux, intensifying IR and metabolic dysfunction in peripheral tissues, such as the liver and skeletal muscle [55, 56]. This intricate immune‐metabolic network not only amplifies the pathological processes associated with obesity but also highlights macrophages as promising therapeutic targets for mitigating obesity‐related metabolic disorders.

## 4 Mechanisms of ATMs Polarization, Accumulation, and Metabolic Reprogramming in Obesity

**Figure 2:**
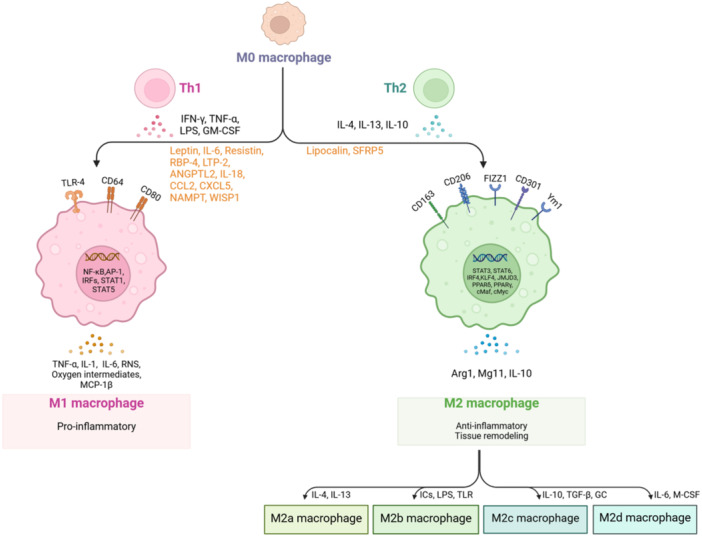
Macrophage polarization: Schematic overview of the major upstream stimuli, signaling pathways, representative markers, and functional features of M1 and M2 macrophages in obesity. ANGPTL2, angiopoietin‐like protein 2; AP‐1, activator protein 1; Arg1, arginase 1; CCL2, C‐C motif chemokine ligand 2; CD, cluster of differentiation; CXCL5, C‐X‐C motif chemokine ligand 5; FIZZ1, found in inflammatory zone 1; GC, glucocorticoids; GM‐CSF, granulocyte‐macrophage colony‐stimulating factor; ICs, immune complexes; IFN‐γ, interferon‐γ; IL, interleukin; IRFs, interferon regulatory factors; JMJD3, jumonji domain‐containing protein 3; KLF4, Krüppel‐like factor 4; LPS, lipopolysaccharide; LTP‐2, lipid transfer protein 2; MCP‐1, monocyte chemoattractant protein‐1; Mgl1, macrophage galactose‐type lectin 1; NAMPT, nicotinamide phosphoribosyltransferase; NF‐κB, nuclear factor kappa B; PPAR, peroxisome proliferator‐activated receptor; RBP‐4, retinol‐binding protein 4; RNS, reactive nitrogen species; SFRP5, secreted frizzled‐related protein 5; STAT, signal transducer and activator of transcription; TGF‐β, transforming growth factor‐β; TLR‐4, toll‐like receptor 4; TNF‐α, tumor necrosis factor‐α; WISP1, WNT1‐inducible signaling pathway protein 1; Ym1, chitinase‐like protein 3.

Macrophages exhibit high plasticity and can undergo phenotypic switching in response to different microenvironmental stimuli and signals in WAT. These stimuli drive macrophages to adopt specific phenotypes and functions distinct from resting macrophages (M0), a process referred to as “macrophage polarization” [34, 57, 58, 59, 60]. In the WAT of obese individuals, macrophages polarize into two major states: M1 (pro‐inflammatory) and M2 (anti‐inflammatory). M1 macrophages produce pro‐inflammatory cytokines and mediators that exacerbate inflammation, whereas M2 macrophages exert anti‐inflammatory effects, promoting tissue repair and homeostasis [61] (Figure 2). In obesity, the shift in macrophage polarization from anti‐inflammatory M2 towards pro‐inflammatory M1 phenotypes, coupled with their expansion and metabolic reprogramming, drives chronic AT inflammation, ultimately exacerbating IR and metabolic dysfunction.

### 4.1 Macrophage Polarization

The polarization of M1 macrophages is typically driven by Th1 cytokines through two major signaling pathways: one mediated by interferon‐γ (IFN‐γ) and TNF‐α, and the other induced by bacterial lipopolysaccharide (LPS) and granulocyte‐macrophage colony‐stimulating factor (GM‐CSF) [57]. The binding of IFN‐γ to its receptor (IFNGR) recruits Janus kinase 1 (Jak1) and Jak2, leading to the activation of interferon regulatory factors (IRF1 or IRF8). This pathway regulates the expression of specific genes, including cytokine receptors (e.g., IL‐6R, IL‐2RA, IL‐15RA), cell activation markers (e.g., CD38, CD69, CD97), and cell adhesion molecules (e.g., ICAM‐1, integrin αL, mucin‐1). Subsequently, macrophages secrete TNF‐α, which further enhances M1 polarization via TNFR1 signaling [62, 63, 64, 65]. M1 macrophages are identified by surface markers such as CD64 and CD80 and are characterized by the production of pro‐inflammatory cytokines, including TNF‐α, IL‐1, IL‐6, reactive nitrogen and oxygen intermediates, IL‐1β, and monocyte chemotactic protein (MCP)‐1β [66, 67, 68]. These cytokines drive inflammation, thereby exacerbating IR [58, 69, 70, 71, 72, 73].

M2 macrophages are polarized through activation of STAT6 via the IL‐4 receptor alpha in response to Th2 cytokines such as IL‐4 and IL‐13. Additionally, IL‐10 controls M2 polarization by activating STAT3 via the IL‐10 receptor [57]. M2‐polarized macrophages are characterized by the expression of markers such as CD163, CD206, CD301, arginase‐1 (Arg1), Mgl1, and IL‐10; they also secrete cytokines and exhibit anti‐inflammatory and tissue‐remodeling functions [30, 58, 61, 71, 72, 74]. M2 macrophages are primarily associated with anti‐inflammatory responses and tissue remodeling, expressing molecules like those found in inflammatory zone 1 (Fizz1), Arg1, chitinase‐3‐like protein‐1 (Ym1), IL‐10, and mannose receptor C‐type 1 (Mrc1/CD206). These molecules are involved in processes such as parasite invasion, tissue remodeling, tumor progression, and the promotion of insulin sensitivity [72, 73].

Prolonged high fructose diet intake has been associated with an increase in M1‐like macrophages, with their numbers rising in proportion to the duration of fructose consumption. The mechanism is that in fat cells, the secretion of leptin and IL‐6 increases. The rise in leptin levels leads to increased lipid accumulation in the liver, while the mRNA levels of proteins within fat cells decrease, causing the fat cells to enlarge. This triggers macrophages and fat cells to release pro‐inflammatory cytokines, which inhibit fat synthesis, ultimately promoting the development of IR and the formation of inflammatory macrophages, particularly M1‐type macrophages [75]. In contrast, moderate‐intensity centrifugal exercise combined with a low‐fat diet reduced inflammatory infiltration and significantly reduced the volume of adipocytes in mice, thereby significantly inhibiting M1 macrophage polarization and promoting M2 macrophage activation [76].

Recent research has increasingly focused on the cytokines, adipokines, proteins, and signaling pathways that influence M1/M2 polarization of ATMs. For example, in HFD‐induced obesity, treatment with IL‐20 monoclonal antibody 7E reduced body weight, improved glucose tolerance and insulin sensitivity, decreased local inflammation, and reduced the number of M1‐like macrophages in AT [71]. Similarly, a study found that in the case of SCAP deficiency, SREBP‐1a activation leads to a decrease in cholesterol outflow downstream of 25‐hydroxycholesterol‐dependent liver X receptor α activation, resulting in an increase in M1 macrophages. Accordingly, the activation of the SCAP‐SREBP‐1a pathway in macrophages could improve obesity by regulating cholesterol homeostasis in ATMs [77].

Emerging areas of interest include the roles of exosomes and gene regulation in fat ATMs polarization. For instance, IgE deficiency influences macrophage polarization [78]. Specifically, IgE deficiency reduces inflammatory cytokine and chemokine production, including IL‐6, IFN‐γ, and MCP‐1, and alters macrophage profiles by decreasing the expression of M1 macrophage markers, increasing the expression of M2 markers, regulating macrophage sterol‐reactive network gene expression, and affecting foam cell formation. Similarly, a study identified six microRNAs (miRNAs) (including the upregulation of miR‐511‐5p and miR‐212‐5p and the downregulation of miR‐142‐3p, miR‐31‐5p, miR‐1894‐3p, and miR‐7b‐5p) associated with macrophage polarization after exercise, linking exosomal changes to ATM polarization. In this study, mice subjected to 8 weeks of exercise exhibited a significant increase in M2 macrophages (CD11c−/CD206+) from 21.0% to 34.8%, while M1 macrophages (CD11c+/CD206−) decreased from 37.6% to 29.3% [79].

In summary, understanding and regulating the polarization of ATMs by cytokines, adipokines, proteins, and signaling pathways, looking into the roles of exosomes and gene regulation, particularly promoting ATMs to transition toward M2‐like macrophages, represents a promising therapeutic target for treating obesity and its associated metabolic disorders.

### 4.2 Accumulation of Macrophages and Efferocytosis

ATM recruitment and infiltration in obesity are closely associated with MCPs, particularly MCP‐1/CCL2 and its receptor CCR2, as well as other MCP family members such as MCP‐2/CCL8, MCP‐3/CCL7, and MCP‐5/CCL12. Recent studies have highlighted that MCP‐1/CCL2 and its receptor CCR2, alongside other chemokines such as macrophage FAP, are involved in the recruitment of inflammatory monocytes to obese AT [80]. Furthermore, IL‐20 has been shown to enhance macrophage retention and monocyte chemotaxis, which in turn contributes to the significant increase in ATM numbers in obese AT [81, 82, 83, 84].

Both migration and local proliferation of macrophages are critical contributors to ATM accumulation in obesity. The local proliferation of resident macrophages plays a crucial role in obesity‐associated inflammation, with the IL‐4/STAT6 pathway identified as a key driver of ATMs proliferation [85]. Early in the progression of obesity, local proliferation of resident macrophages plays a pivotal role in increasing ATM numbers, and there is no significant increase in CCL2 production at this stage [20]. As obesity progresses, the migration of monocytes to AT becomes more significant, and migrating macrophages subsequently proliferate locally [85]. These proliferating ATMs predominantly accumulate in CLS and are closely associated with efferocytosis, and recently showed that obesity‐associated macrophages propagate mitochondrial fragmentation signals to adipose stem cells, inducing ferroptosis and driving visceral fat dysfunction in a TREM2^+^ ATM–dependent manner [86]. Osteopontin has been shown to facilitate both macrophage recruitment and proliferation in this process [87]. Notably, obesity‐induced local macrophage proliferation drives islet inflammation without the need for monocyte recruitment [88]. In contrast, inhibiting GSK3 reduced macrophage apoptosis inhibitor production, partially through STAT3 inactivation, which decreased FFA and chemokine levels in VAT, inhibiting macrophage and monocyte migration, reducing M1 macrophage levels, increasing M2 macrophages, and diminishing circulating inflammatory monocytes [89].

Adipocyte death, both apoptotic and necrotic, is a central cause of inflammation in obese AT [90]. Once adipocytes reach a critical size threshold, they undergo efferocytosis mediated by resident ATMs, which is essential for maintaining tissue homeostasis and resolving inflammatory responses [20]. However, when adipocytes surpass this threshold, resident macrophages become pro‐inflammatory, and efferocytosis becomes impaired, leading to hyper‐inflammatory responses [54]. This failure to clear dead adipocytes contributes to chronic inflammation, which is a key driver of obesity pathogenesis, ultimately resulting in systemic inflammatory responses and AT dysfunction [91, 92].

### 4.3 Adipokines

AT releases molecules, known as adipokines, with either pro‐inflammatory or anti‐inflammatory bioactivity that regulate AT inflammation and metabolism [93]. The role of adipokines in modulating monocyte recruitment, macrophage polarization, and immune inflammation has garnered considerable attention in recent years. In diet‐induced obesity, the upregulation of pro‐inflammatory adipokines outpaces that of anti‐inflammatory adipokines, contributing to obesity‐related metabolic dysfunction. Several key adipokines have been identified, including pro‐inflammatory adipokines such as leptin, TNF, IL‐6, resistin, RBP4, lipid transport protein 2 (LTP2), ANGPTL2, IL‐18, CCL2, CXCL5, and NAMPT, as well as anti‐inflammatory adipokines like lipocalin and SFRP5 [94]. However, many novel obesity‐related adipokines remain to be fully validated. For instance, Wnt1‐inducible signaling pathway protein‐1 (WISP1) has been shown to promote adipocyte differentiation and stimulate macrophages, leading to pro‐inflammatory responses [95]. Similarly, leucine‐rich α−2‐glycoprotein 1 (LRG1), an obesity‐associated adipokine, selectively binds to the liver, promoting hepatic steatosis by increasing de novo lipogenesis and inhibiting β‐oxidation of fatty acids. Additionally, LRG1 suppresses hepatic insulin signaling by down‐regulating insulin receptor substrates (IRSs) 1 and 2, exacerbating high‐fat diet‐induced hepatic steatosis and IR [96]. Furthermore, accumulating evidence highlights the role of adipokines, including leptin, resistin, and adiponectin, in macrophage polarization, contributing to either inflammatory or anti‐inflammatory effects [97]. Notably, sex differences have been observed in adipokine secretion, with men exhibiting higher levels of leptin than women, which leads to an excess of macrophages in male AT, resulting in elevated inflammatory cytokines, such as TNF‐α [98].

### 4.4 Mitochondrial Energy Metabolism and Metabolic Reprogramming

Mitochondria, as the energy centers of adipocytes, play a central role in key metabolic processes, including ATP production via oxidative phosphorylation (OXPHOS), substrate generation for cellular metabolism (e.g., fatty acid synthesis and oxidation), regulation of lipid turnover (cellular triglyceride homeostasis), adipogenesis, and adipokine secretion [99]. The tricarboxylic acid (TCA) cycle, fatty acid β‐oxidation (FAO), and OXPHOS are critical components of mitochondrial energy metabolism. In macrophages, mitochondrial respiratory function critically influences energy metabolism. For example, IL‐25 has been shown to enhance mitochondrial respiratory capacity, oxygen consumption, and the production of NAD^+^/NADH and ATP [100]. The inflammatory transcription factor IRF5 disrupts the TCA cycle and impairs macrophage mitochondrial respiration in the early stages of glucose intolerance preceding IR [101].

**Figure 3:**
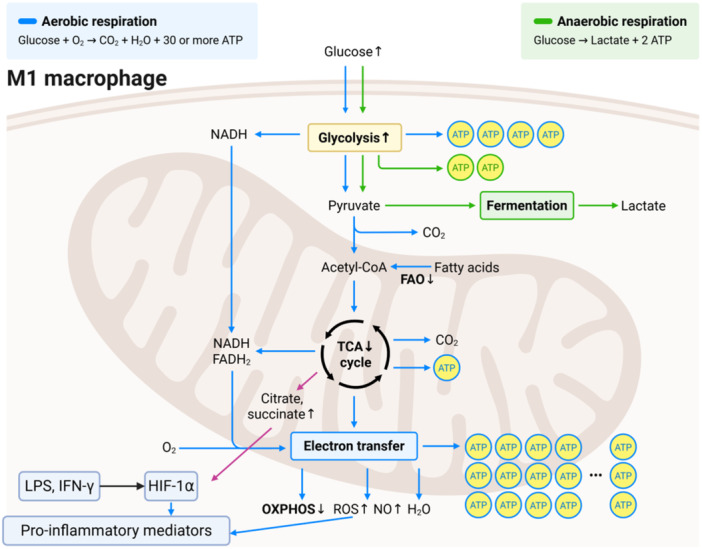
Mitochondrial energy metabolism and metabolic reprogramming. Under stimulation by LPS and IFN‐γ, glycolysis is significantly upregulated, with excess carbon converted to lactate, while FAO and OXPHOS are suppressed. The TCA cycle is disrupted, leading to the accumulation of citrate and succinate, which stabilizes HIF1α and promotes the release of pro‐inflammatory mediators, thereby facilitating M1 polarization. ATP, adenosine triphosphate; CO_2_, carbon dioxide; FADH_2_, reduced flavin adenine dinucleotide; FAO, fatty acid oxidation; HIF‐1α, hypoxia‐inducible factor‐1α; IFN‐γ, interferon‐gamma; LPS, lipopolysaccharide; NADH, reduced nicotinamide adenine dinucleotide; NO, nitric oxide; O_2_, oxygen; OXPHOS, oxidative phosphorylation; ROS, reactive oxygen species; TCA cycle, tricarboxylic acid cycle.

In ATMs, pro‐inflammatory M1 macrophages primarily depend on glycolysis and mitochondrial respiration, whereas anti‐inflammatory M2 macrophages utilize FAO and OXPHOS for ATP production [102, 103]. Pro‐inflammatory stimuli, such as LPS and IFN‐γ, drive M1 macrophage polarization through hypoxia‐inducible factor‐1α (HIF1α) signaling, while concurrently suppressing mitochondrial OXPHOS in M2 macrophages [104, 105]. The reliance on glycolysis in M1 macrophages facilitates the secretion of pro‐inflammatory cytokines, thereby contributing to chronic low‐grade inflammation and metabolic dysregulation. Excess carbon from glycolysis in M1 macrophages is excreted as lactate, while disruptions in the TCA cycle—marked by the accumulation of citrate and succinate—stabilize HIF1α, further promoting M1 polarization [106]. These metabolites amplify pro‐inflammatory responses, perpetuating inflammation and metabolic imbalance (Figure 3).

Reactive oxygen species (ROS) and nitric oxide (NO), byproducts of glycolysis, are significant mediators of inflammatory signaling in macrophages [107]. Downregulation of macrophage autophagy under inflammatory conditions exacerbates ROS accumulation, fostering a localized inflammatory microenvironment [108]. Studies suggest that glutamine supplementation can attenuate glycolysis, reduce pro‐inflammatory gene expression, and downregulate uridine diphosphate N‐acetylglucosamine, although its levels are decreased in obesity [109]. Type I inflammatory cytokines and obesity‐associated molecules (e.g., IFN‐γ, TNF, leptin, insulin, and palmitate) induce PD‐1 expression in macrophages via mTORC1‐ and glycolysis‐dependent pathways, providing negative feedback to inhibit glycolysis [110].

While significant progress has been made in understanding macrophage metabolic reprogramming in obesity, the precise mechanisms and key regulators remain unclear. Future research should focus on identifying critical factors that govern these processes, which could unveil novel therapeutic targets for obesity and its related metabolic disorders.

## 5 The Link Between Obesity and IR

IR is not a disease in itself but rather a physiological abnormality that manifests in conditions such as obesity and diabetes. Insulin, a critical hormone regulating multiple metabolic pathways, plays a pivotal role in various physiological processes throughout the human body. Consequently, IR induced by obesity can disrupt these processes, leading to the onset of numerous disorders. The development and progression of IR are influenced by a complex interplay of multiple factors [111]. To date, substantial research has been conducted to elucidate the mechanisms underlying IR, yielding significant findings. It is well‐established that IR is intricately linked to conditions such as diabetes [112], cancer [113], metabolic syndrome [114], hypertension [115], and obesity [116], among others. This chapter focuses specifically on summarizing the potential mechanisms and interactions between obesity and IR.

### 5.1 Factors Affecting IR

Dysfunctional AT in obese individuals plays a pivotal role in the development of IR, and numerous studies have established a significant correlation between obesity and the onset of IR [117, 118]. Current evidence suggests that hypertrophic adipocytes release FFAs, lipopolysaccharides, ROS, and pro‐inflammatory cytokines, thereby inducing lipotoxicity in non‐ATs. This factors triggers systemic inflammation, disrupts insulin signaling pathways, and ultimately leads to IR [116, 119]. TNF‐α has been shown to promote IR in cardiomyocytes and adipocytes by activating pro‐inflammatory pathways. This impairs insulin signaling at the level of IRS proteins, contributing to IR in the liver and muscle tissues [120]. Interestingly, vitamin C has been reported to mitigate TNF‐α‐induced impairments in glucose uptake and glycogen synthesis by upregulating GLUT2 expression and activating the IRS‐1/AKT/GSK3β pathway, suggesting its potential efficacy in improving IR [121].

In addition to inflammatory mechanisms, genetic factors also influence IR. Maclaren et al. noted a strong familial predisposition to IR, with individuals born small for gestational age exhibiting an elevated risk of IR. The incidence of IR increases significantly during puberty in obese children, particularly in those who gradually progress to metabolic decompensation [122]. Obesity and IR have a bidirectional relationship influenced by age and sex. For instance, a study on obese children found an inverse relationship between childhood IR in girls and the risk of adult obesity, whereas no such association was observed in boys [123].

Hormonal dysregulation is another key contributor to IR in obesity. Both leptin and insulin levels are elevated in obese individuals; these hormones act as satiety signals, modulating food intake and maintaining energy homeostasis [124]. Leptin, in particular, binds to leptin receptors (LEPR) on pancreatic β‐cells, activating ATP‐sensitive K^+^ channels and regulating insulin secretion [125, 126]. Overexpression of leptin promotes the progression of IR, as hyperinsulinemia and IR can upregulate leptin gene expression, creating a feedback loop that exacerbates IR [127]. Similarly, adiponectin directly influences pancreatic β‐cells, regulating insulin secretion and contributing to metabolic dysregulation [128].

Additional factors further exacerbate IR in obese individuals. For example, low serum magnesium levels have been implicated in the development of IR in obese children [129]. Dietary patterns characterized by high energy density, excessive fat and fructose intake, and low fiber and dairy consumption not only promote obesity but also contribute to central nervous system‐related IR [130]. The anatomical distribution of adiposity also influences IR differently based on sex. Research has demonstrated that waist circumference (WC) is a more significant determinant of IR in men, whereas body mass index (BMI) has a greater impact in women [131].

Future studies should focus on elucidating the multifactorial mechanisms underlying IR and its relationship with obesity. A deeper understanding of these factors will inform the development of targeted therapeutic strategies to address IR and its associated complications.

### 5.2 Impact of IR Development After Obesity

IR is intricately linked to various physiological processes in the human body, leading to alterations in the expression levels of numerous biomolecules. Recent research has identified several of these molecules, some of which hold promise as biomarkers for the early detection and progression of IR in obese individuals. STEAP4, a novel anti‐obesity gene, is significantly downregulated in the AT of obese patients [132]. Additionally, the neutrophil‐to‐lymphocyte ratio is markedly elevated in obese individuals with IR, serving as a potential indicator of systemic inflammation [133]. PM20D1, a circulating biomarker, exhibits a strong association with obesity, IR, and metabolic syndrome, making it a viable candidate for clinical diagnosis [134].

Spexin and kisspeptin, two novel peptides implicated in metabolism, appetite regulation, puberty, and reproductive function, have been shown to be reduced in female obese patients, and their levels are negatively correlated with the IR index and hormones influencing female insulin sensitivity [135]. Furthermore, IR has demonstrable effects on nerve conduction in obese children. Studies reveal that the nerve conduction velocity of the motor median nerve is significantly higher in obese children with IR compared to those without IR, though both are lower than in healthy controls [136]. These findings provide valuable insights into potential interventions for nerve conduction disorders in obese children.

Moreover, investigations into pre‐pubertal obesity have identified 15 specific circulating miRNAs with significant dysregulation, offering potential for early detection of metabolic abnormalities in obese children [137]. Among these, circulating miR‐122 is elevated in obese patients and positively correlates with IR, further highlighting its diagnostic value [138]. The progression of IR significantly increases the risk of chronic conditions such as chronic kidney disease [139], peripheral vascular disease [140], hypertension [115], and osteoporotic fractures [141]. Notably, brain IR in obesity has been linked to defects in neuroplasticity. Emerging evidence suggests that exercise interventions can ameliorate these impairments by enhancing brain insulin signaling and regulating mitochondrial quality control [141].

Given the extensive impact of IR on metabolic and systemic health, the urgent need arises to devise effective strategies to alleviate IR in obesity, thereby reducing associated comorbidities and improving overall quality of life.

### 5.3 Macrophages Involved in the Process of IR

In obese individuals, the activation phenotype of macrophages in AT shifts from the M2 polarization state that protects adipocytes and suppresses inflammation to a pro‐inflammatory M1 state [30]. ATMs are mainly pro‐inflammatory M1 macrophages, excessively secreting inflammatory cytokines and chemokines, including TNF‐α, IL‐6, IL‐1β, MCP‐1, CCL2, and macrophage migration inhibitory factor [142]. Numerous studies confirm that inflammatory cytokines and chemokines secreted by M1 macrophages further promote local inflammatory responses and affect gene expression in adipocytes, leading to systemic IR [83].

Notably, TNF‐α secreted by macrophages blocks insulin action in adipocytes by downregulating glucose transporter 4 (GLUT4) and IRS‐1, while TNF‐α neutralizing antibodies partially reverse the induced IR [143]. Studies show that abnormal secretion of Th2 cytokines by adipocytes in obese individuals inhibits PPARδ pathway activation, hinders macrophage polarization toward the anti‐inflammatory M2 phenotype, reduces GLUT4 expression, decreases insulin‐stimulated glucose uptake, and exacerbates IR [144, 145]. In obese patients with a high‐fat diet, elevated FFAs activate the TLR4 pathway in ATMs. Secreted pro‐inflammatory factors like TNF‐α inhibit AMPK activity by upregulating PP2C, reduce skeletal muscle FAO, promote lipid accumulation, and then phosphorylate IRS‐1 via kinases such as protein kinase C and c‐Jun N‐terminal kinase, triggering IR and forming an “inflammation‐metabolic disorder” vicious cycle [146].

In addition, a study found that obese patients have significantly elevated serum IL‐29 levels, which reduce glucose uptake and insulin sensitivity by decreasing GLUT4 and AKT signaling, while upregulating IL‐1β, IL‐8, and MCP‐1 expression in human adipocytes [147]. Further, high expression of IRF5, reversibly induced by inflammatory stimuli, promotes M1 macrophage polarization [148]. Experimental results show that mice lacking IRF5 exhibit accumulation of M2 macrophages in epididymal WAT, higher collagen deposition limiting adipocyte size, and enhanced insulin sensitivity, indicating an inverse correlation between IRF5 and insulin sensitivity [149].

### 5.4 Treatment Strategies Inspired by IR

Various treatment strategies for improving IR have been identified to date, although many of these still require extensive validation in clinical practice to confirm their effectiveness and safety. A study in rats demonstrated that electroacupuncture could significantly enhance insulin sensitivity by modulating the glucagon‐like peptide‐1 (GLP‐1) pathway, influencing the dopamine neural reward circuit in the ventral tegmental area and the nucleus of the solitary tract [150]. Research involving obese and lean men revealed that regular resistance training improved insulin sensitivity in the lean group; however, for obese individuals, surpassing a certain threshold, resistance training alone proved insufficient to ameliorate IR, necessitating additional interventions such as caloric restriction [118]. In patients with early‐stage IR, endothelin‐1 antagonists have shown potential in enhancing glucose tolerance and mitigating the progression of IR [151]. Clusterin has been implicated in improving IR by facilitating leptin transport across the blood–brain barrier and binding to the LRP2 receptor, which sensitizes LEPR in the hypothalamus [152]. Furthermore, in obese mice, the expression of miR‐181b is reduced in endothelial cells of AT. Modulating the expression of miR‐181b in this context has been shown to improve endothelial function in WAT, thereby enhancing glucose homeostasis and insulin sensitivity [153]. Although numerous effective strategies for improving IR exist, further research is warranted to explore additional therapeutic possibilities for obese patients.

## 6 The Relationship Between Obesity and Fatty Acid Metabolism

**Figure 4:**
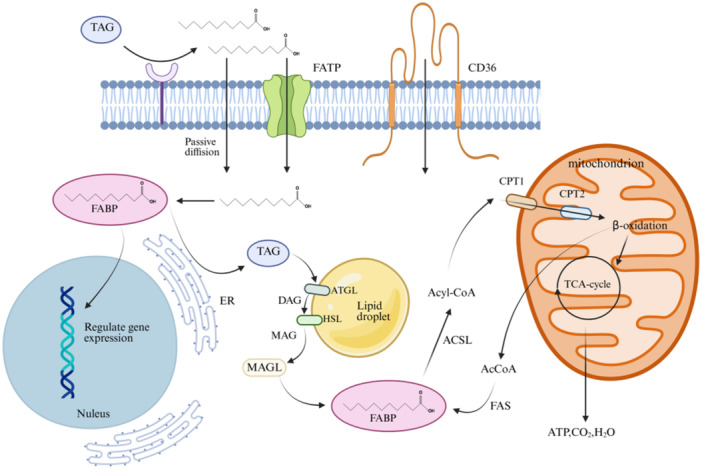
The process of fatty acid metabolism in obesity. TAG are sequentially hydrolyzed by ATGL, HSL, and MAGL to release FFAs, which enter cells via CD36/FATP and are intracellularly shuttled by FABP. FFAs are then activated by ACSL to acyl‐CoA and transported into mitochondria by CPT1/CPT2, where β‐oxidation and the TCA cycle produce ATP. AcCoA, acetyl coenzyme A; ACSL, acyl‐CoA synthetase long‐chain family member; Acyl‐CoA, acyl coenzyme A; ATGL, adipose triglyceride lipase; ATP, adenosine triphosphate; CD36, cluster of differentiation 36; CO_2_, carbon dioxide; CPT1, carnitine palmitoyltransferase 1; CPT2, carnitine palmitoyltransferase 2; DAG, diacylglycerol; ER, endoplasmic reticulum; FABP, fatty acid‐binding protein; FAS, fatty acid synthase; FATP, fatty acid transport protein; MAG, monoacylglycerol; MAGL, monoacylglycerol lipase; TAG, triacylglycerol; TCA cycle, tricarboxylic acid cycle.

Obesity is a complex, chronic condition characterized by excessive fat accumulation and/or abnormal fat distribution in the body, leading to weight gain and potentially detrimental health effects. As such, understanding the metabolism of fat in the human body is crucial for the effective treatment of obesity. Adipocytes, the primary cells in AT, play a central role in energy storage and are closely connected to other organs, performing various physiological functions [154]. However, these biological functions are significantly altered in obesity. Nearly all individuals with obesity experience disturbances in fat metabolism and IR, which are particularly evident in the increased mass of AT and the accumulation of TAG in muscle cells. The key aspects of fatty acid metabolism, which include lipolysis, fatty acid uptake, activation, esterification, and oxidation, are of particular focus (Figure 4). Investigating the relationship between obesity and fatty acid metabolism can provide valuable insights and potential therapeutic strategies for combating obesity.

### 6.1 Breakdown of TAG

Adipose triglyceride lipase (ATGL), a critical enzyme in lipid metabolism, catalyzes the hydrolysis of TAG into diacylglycerol (DAG) and FFAs [155]. As a central player in fat mobilization, ATGL is pivotal for maintaining energy homeostasis and regulating fat metabolism [156]. Its expression is ubiquitous across multiple tissues, with the highest levels observed in AT [157, 158, 159]. The activity of ATGL is influenced by various factors, including hormones, protein interactions, and transcriptional regulation. Regulatory proteins, such as perilipins [160, 161], cell death activator CIDE‐3 [162], comparative gene identification‐58 (CGI‐58) [163], and G0/G1 switch protein 2 [164], have been shown to exert significant effects on ATGL. Additionally, ATGL has been implicated in SIRT1‐mediated autophagy/lipophagy, controlling hepatic lipid droplet catabolism and FAO [165].

Hormone‐sensitive lipase (HSL) is another essential lipolytic enzyme that hydrolyzes TAG, yielding FFAs and glycerol during catecholamine‐regulated lipolysis [166]. HSL exhibits broad substrate specificity, including TAG, DAG, monoacylglycerol (MAG), and cholesterol esters, with a particular preference for DAG [167]. It further hydrolyzes DAG to MAG, which is subsequently broken down by monoacylglycerol lipase (MAGL) to produce glycerol and release the final FFA [168].

Conflicting evidence exists regarding the expression of ATGL and HSL in obesity, with no consensus in the literature. Some studies report significantly reduced gene expression levels of ATGL and HSL in obese individuals, suggesting that diminished catecholamine‐induced lipolysis and reduced HSL expression may constitute primary defects in obesity [169, 170]. Conversely, De Naeyer et al. [171] observed increased mRNA levels of ATGL and HSL in morbidly obese men, although these increases were not reflected in protein expression or phosphorylation levels. Notably, both ATGL and HSL are insulin‐sensitive, potentially linking their activity to the IR commonly observed in obese individuals [172].

While beta‐3 adrenergic receptors are known to mediate lipolysis in human white adipocytes, their role in obesity remains controversial [173]. Evidence indicates that beta‐3 adrenergic receptor agonists fail to elicit significant lipolytic effects in obese patients, raising questions about the functional relevance of these receptors in adipocytes [174]. Additionally, Jocken et al. demonstrated impaired lipolytic responses in the abdominal SAT of obese individuals during intravenous infusion of the non‐selective beta agonist isoproterenol. Further research is needed to elucidate the relationship between lipolysis and obesity [175].

Lipoprotein lipase (LPL) is another enzyme involved in lipid metabolism, facilitating the hydrolysis of triglyceride‐rich lipoproteins (TRL) [176]. Despite its importance, the tissue‐specific regulation of LPL remains poorly understood. Physiological factors modulate LPL activity, and a diet high in saturated fats and refined carbohydrates has been shown to increase plasma lipid levels and obesity by altering LPL and VLDL receptor activity while reducing TRL clearance [177]. Given the limited research on the role of LPL in obesity, future studies should focus on elucidating its mechanisms and impact in this context.

### 6.2 Fatty Acid Uptake

The cellular uptake of fatty acids requires the involvement of specific transport proteins, several of which have been extensively characterized and studied, including fatty acid translocase (FAT) (CD36), fatty acid transport proteins (FATPs), and fatty acid‐binding proteins (FABPs). These proteins play critical roles in facilitating the efficient uptake, intracellular trafficking, and metabolism of fatty acids, ensuring their availability for energy production, lipid synthesis, and other physiological processes. Understanding the regulation and function of these transport proteins is essential for elucidating their contributions to metabolic homeostasis and their potential roles in obesity and related metabolic disorders.

#### 6.2.1 CD36/FAT

CD36, also known as FAT and glycoprotein IIIb, is a member of the class B2 scavenger receptor family [178]. It is widely expressed in various tissues, including endothelial cells, cardiomyocytes, renal tubular epithelial cells, hepatocytes, adipocytes, platelets, and macrophages [179]. As a receptor for long‐chain fatty acids (LCFAs), CD36 plays a crucial role in fatty acid metabolism [180].

Numerous studies have demonstrated that the expression and levels of CD36 are influenced by obesity. For instance, one study reported a correlation between obesity and elevated levels of FFAs [181]. Overexpression of CD36 has been implicated in several adverse outcomes associated with obesity. Analysis of SAT and VAT from lean, overweight, and obese individuals, as well as patients with type 2 diabetes (T2D), revealed that FAT/CD36 expression was significantly upregulated. This upregulation correlated with BMI and circulating levels of glucose and insulin [182]. Additionally, CD36 overexpression in obese patients has been linked to impaired granulosa cell function [183].

The role of CD36 in obesity‐related inflammation is particularly notable. Studies have shown that CD36 expression is induced in preadipocytes of obese patients and in HFD mice, which is accompanied by lysosomal dysfunction [184]. Furthermore, research by Douglas Braymer et al. on obesity‐prone (OP) and obesity‐resistant (OR) rats demonstrated dysregulation of lingual CD36 mRNA expression in OP rats, and the dysregulation likely contributes to increased fat intake and heightened susceptibility to obesity [185].

#### 6.2.2 FATPs

FATPs comprise a family of six highly homologous isoforms that are ubiquitously expressed in tissues involved in fatty acid utilization [186]. FATPs play a pivotal role in lipid uptake and metabolism and work in concert with CD36 to regulate the uptake of LCFAs [187].

Accumulating evidence from animal studies highlights alterations in FATP expression levels in response to obesity. For example, a study on prepartum obese dairy cows revealed that increased lipin‐1 expression and decreased FATP1 expression in skeletal muscle partially contributed to IR [188]. Similarly, in a study of pregnant rats subjected to an HFD, obesity significantly reduced the mRNA and protein expression levels of CD36, FATP1, and FATP4 in the placenta [189]. In contrast, maternal obesity in obese ewes was associated with increased subcutaneous fat thickness in their fetuses, accompanied by elevated mRNA expression of CD36, FATP1, and FATP4 in fat depots, suggesting an increased risk of offspring obesity [190].

Further insights come from a study on genetically obese rats, which demonstrated a tissue‐specific increase in oleic acid uptake in adipocytes and elevated levels of FATP mRNAs [191]. Additionally, other studies have reported increased FATP levels in cardiomyocytes and skeletal muscles, with the content of FATP1 protein rising in the soleus muscle but declining in the gastrocnemius muscle in obese mouse models [192, 193]. However, further research is warranted to elucidate the specific mechanisms underlying the regulation of FATP expression and its role in obesity.

#### 6.2.3 FABPs

FABPs comprise small, water‐soluble proteins that bind LCFAs and other bioactive ligands to facilitate their intracellular transport and localization. To date, 12 FABP family members have been identified, 10 of which are expressed in humans, displaying distinct tissue‐specific expression patterns [194]. Among these, dysregulation of FABP4 is strongly associated with various human diseases, including diabetes and obesity. Also known as adipocyte FABP or adipocyte protein 2, FABP4 is located on chromosome 8q21 and exhibits a conserved tertiary structure characteristic of the FABP family [195]. FABP4 is predominantly expressed in WAT and BAT, as well as in monocytes and macrophages [196].

Elevated levels of FABP4 have been observed in individuals with obesity, where excessive dietary intake of saturated fatty acids can directly upregulate FABP4 expression, leading to the production of cytotoxic ceramides and macrophage apoptosis [197]. Additionally, FABP4 has been implicated in the progression of malignant tumors, including breast, prostate, and ovarian cancers [197, 198, 199]. As a critical component of fatty acid metabolism, FABPs are gaining increasing attention in biomedical research. Future studies are needed to further elucidate their roles in various diseases, with the aim of uncovering novel therapeutic strategies and insights for disease management.

### 6.3 Fatty Acid Activation

After entering cells, fatty acids are converted into fatty acyl‐CoA through the catalytic action of long‐chain acyl‐CoA synthetase (ACSL). This conversion enhances the water solubility of fatty acids and transforms them into high‐energy compounds, enabling their utilization in metabolic processes such as mitochondrial FAO.

ACSL is an enzyme family responsible for catalyzing the conjugation of fatty acids with coenzyme A. This family comprises five members—ACSL1, ACSL3, ACSL4, ACSL5, and ACSL6—each exhibiting distinct substrate specificities [200]. Certain ACSL subtypes are closely associated with obesity. For instance, obese animals with cardiomyopathy have been shown to exhibit increased cardiac ACSL1 levels and decreased GLUT1 levels [201]. Consistently, Yang et al. reported that sortilin‐dependent enrichment of ACSL1 in adipocyte mitochondria blunts UCP1‐mediated thermogenesis and reduces whole‐body energy expenditure in diet‐induced obese mice [202]. And a study demonstrated that aerobic exercise increased the abundance of ACSL6 protein while reducing ACSL4 levels. Notably, elevated skeletal muscle ACSL6 was associated with increased intramyocellular fat, a finding with potential therapeutic implications for obesity [203]. In another study, ACSL5 overexpression was observed in the myocytes of obese women, which elevated mitochondrial superoxide production and led to significant reductions in insulin‐stimulated p‐Akt and p‐AS160 protein levels, ultimately impairing insulin signaling [204]. Additionally, studies on ob/ob mice revealed upregulated ACSL activity in the liver and AT. Similar findings were reported in rats fed an HFD [205, 206]. These results highlight the role of ACSL in obesity and its potential as a target for therapeutic intervention.

### 6.4 FAO in Mitochondria

Fatty acids from extracellular sources undergo oxidation within mitochondria, generating ATP. LCFAs enter mitochondria via the carnitine shuttle system, while short‐chain fatty acids can diffuse freely across the mitochondrial membrane. A study on medium‐chain fatty acids revealed that their oxidation in the liver is uncoupled from carnitine and occurs independently, with a similar, albeit lesser, effect observed in the kidneys. However, this process is not applicable to the heart or skeletal muscle [207]. Numerous studies have explored FAO levels in obese individuals, yet significant discrepancies exist among the findings, and a definitive, unified conclusion has yet to be reached. Some research indicates an increase in FAO in the muscles of rats on a high‐fat diet, obese Zucker rats, and db/db mice [208]. In contrast, other studies suggest a reduction in fatty acid metabolism in obese individuals [209]. These conflicting results underscore the urgent need for further investigation to elucidate changes in FAO levels in obese patients.

### 6.5 The Impact of Macrophages on Fatty Acid Metabolism

As a key hub linking metabolism and immunity, macrophages significantly influence the lipid metabolic network in obese organisms by finely regulating processes such as fatty acid uptake and inflammatory signaling.

Under obesity, macrophage infiltration in AT increases, and the imbalance between Th2 cytokines (IL‐13/IL‐4) and Th1 cytokines (IFN‐γ, TNF‐α) may inhibit PPARδ pathway activation. PPARδ deficiency leads to macrophage‐secreted TNF‐α downregulating perilipin expression, allowing HSL to access lipid droplets more readily and accelerate lipolysis [145]. Pro‐inflammatory factors such as TNF‐α and IL‐1β secreted by macrophages induce NLRP3 inflammasome activation in adipocytes, interfere with PPARγ expression, promote lipolysis, inhibit fatty acid synthesis and storage, facilitate FFA release, and exacerbate lipotoxicity [210]. Studies have shown that TNF‐α can promote lipolysis by downregulating the expression of lipolysis‐related molecules (e.g., adiponectin, ATGL, HSL), while inducing proteasomal degradation of PPAR‐γ to reduce its binding to the promoter of G0/G1 switch gene 2 (G0S2), thereby decreasing G0S2 expression [211]. Research indicates that TNF‐α treatment induces significant morphological changes in mitochondria of 3T3‐L1 adipocytes and significantly reduces ATP production compared with the control group, affecting mitochondrial FAO [212]. IL‐6, a cytokine secreted by macrophages, plays a crucial role in immunity and metabolism. Analysis of human skeletal muscle cells has shown that IL‐6 increases glycogen synthesis via a PI3 kinase‐dependent mechanism and enhances lipid oxidation via an AMPK‐dependent mechanism in skeletal muscle [213].

Furthermore, macrophages also directly participate in lipid phagocytosis and metabolism. Scavenger receptors, such as CD36, secreted by macrophages (mentioned above), mediate excessive uptake of LCFAs by adipocytes in obesity, leading to intracellular lipid accumulation and subsequent inflammatory progression [184, 214].

### 6.6 Treatment Schemes Inspired by Fatty Acid Metabolism

Fatty acid metabolism plays a crucial role in the regulation of fat degradation and synthesis. Numerous studies have explored various treatment strategies for obesity, focusing on different aspects of metabolic pathways. Dietary compounds, in particular, have been shown to influence fat degradation. For instance, calcium supplementation has been found to aid in weight loss in obese mice and human subjects on energy‐restricted diets, primarily by inhibiting parathyroid hormone secretion, which stimulates fat breakdown [215]. Caffeine consumption promotes weight loss and weight maintenance by enhancing thermogenesis and fat degradation [216]. Short‐term ethanol intake also facilitates fat breakdown; however, chronic ethanol consumption inhibits the phosphorylation of HSL, a key enzyme in lipolysis, thereby reducing fat degradation [217]. Furthermore, increasing fat breakdown can be achieved through hormonal regulation. In conditions of negative energy balance, such as fasting, starvation, or prolonged exercise, the lipolytic sensitivity to catecholamines is heightened, which enhances fat cell lipolysis and can lead to sustained fat loss [218, 219]. Additionally, studies in obese rats have shown that treatment with green tea catechins can prevent obesity‐induced kidney damage by modulating the expression of CD36 and PPARγ in renal tissues [220].

Manipulating the expression of key proteins involved in fatty acid metabolism presents a promising approach for weight management. Targeting CD36, for example, may prove to be an effective strategy to ameliorate metabolic dysfunction in obese individuals [221]. A genetic variation in the promoter region of the human A‐FABP gene has been identified, which reduces the transcriptional activity of the aP2 promoter, leading to decreased FABP expression in AT [222]. Individuals carrying this variation exhibit lower serum TAG levels, a reduced risk of coronary heart disease and T2D, and a decreased likelihood of developing T2D in obese populations [223]. Moreover, semaglutide, a GLP‐1 analog, has been shown to reduce the expression of lipid metabolism‐related proteins such as CD36, FABP5, ACSL, and LPL, effectively improving visceral fat accumulation and glucose intolerance [224]. In another study, overexpression of ACSL1 in C2C12 myotubes increased FAO and improved IR, highlighting the potential of targeting ACSL1 to mitigate IR in obesity [225].

In summary, in‐depth research on fatty acid metabolism holds significant promise for improving obesity and its associated IR. However, many underlying mechanisms remain to be elucidated, warranting further investigation in the future.

## 7 The Role of Gut Microbiome During Obesity

The gut microbiome is a critical component of the human metabolic system and plays a pivotal role in maintaining host health. It contributes to immune regulation, angiogenesis, and neural function through the production of vitamins, nutrient absorption, and secretion of bioactive small molecules [226]. The gut microbiota is closely linked to the pathogenesis of various systemic diseases, ranging from inflammatory bowel disease and colorectal cancer to neurological disorders such as Parkinson's disease, autism, and anxiety [227, 228, 229, 230, 231]. Despite significant advancements in understanding its influence on intestinal and neurological conditions, the mechanisms underlying disease development remain highly complex. The intricate interactions between the gut microbiome and the human host underscore the challenges in elucidating its role in disease progression. This section seeks to explore the relationship between the gut microbiome and human obesity, shedding light on potential mechanisms and discussing prospects for utilizing microbiome‐targeted strategies in obesity treatment.

### 7.1 The Composition of the Gut Microbiome

The gut microbiome is highly sensitive to environmental changes and is influenced by various factors, including calorie intake and environmental humidity. A study utilizing a conditional housing male mouse model demonstrated that exposure to a humid and high‐temperature environment disrupted gut microbiota composition, such as a reduction in the abundance of *Lactobacillus murinus*, and increased serum secondary bile acid levels. These changes were associated with anxiety‐like behaviors in male mice. Similarly, human samples collected during humid and hot seasons also exhibited decreased *L. murinus* abundance and elevated serum lithocholic acid concentrations [231].

However, diet has been shown to exert a more profound influence on gut microbiota composition, particularly through changes in food intake and the physiological state of the digestive system [232, 233]. For instance, in a study conducted by David et al. [234], 10 volunteers were divided into two dietary groups: a “plant‐based diet” (rich in grains, legumes, fruits, and vegetables) and an “animal‐based diet” (comprising meat, eggs, and cheese), while maintaining equivalent caloric intake, the animal‐based diet was observed to exert a more pronounced impact on the composition of the gut microbiota compared to the plant‐based diet over a 5‐day period.

In addition to diet and environmental factors, the gut microbiome is influenced by circadian rhythms—endogenous processes that regulate cellular, organ, and systemic functions over a 24‐h cycle and are observed across various organisms, including bacteria, fungi, plants, and animals [235]. Cyanobacteria, for instance, display distinct circadian fluctuations. Similarly, the gut microbiome has been shown to exhibit endogenous circadian rhythms. A study investigating the impact of day‐night cycles on mouse fecal microorganisms revealed significant diurnal variations in the composition of the gut microbiota [236].

This evidence highlights the complex interplay between environmental factors, diet, and circadian rhythms in shaping the gut microbiome, emphasizing the need for further research to understand these dynamics and their implications for host health.

### 7.2 The Relationship Between Gut Microbiome, Diet, and Obesity

Numerous studies have demonstrated the inextricable link between obesity and the gut microbiome, as well as the impact of factors such as diet. The gut microbiome plays a pivotal role in the development of obesity. Germ‐free mice, for instance, do not exhibit signs of obesity or metabolic dysfunction. However, when the gut microbiome from obese mice is transplanted into these germ‐free mice, the latter promptly display obesity‐related traits [237]. Additionally, animal models have shown that the gut microbiome can contribute to obesity through the gut–brain axis [238].

However, the composition of the gut microbiome in obese individuals is not uniform. Research indicates that, compared to healthy individuals, obese patients exhibit distinct microbial profiles, with variations corresponding to the degree of obesity [239]. For example, the relative proportion of Bacteroidetes is reduced in obese individuals, while the abundance of Firmicutes remains unchanged [240]. Consequently, the Firmicutes‐to‐Bacteroidetes ratio may serve as a potential marker for obesity susceptibility [241]. A study on energy balance revealed that a 20% increase in Firmicutes proportion is associated with an additional 150 kcal of nutrient absorption, whereas a similar increase in Bacteroidetes correlates with a 50 kcal reduction in absorption [242]. Furthermore, when comparing obese and lean twins, it was found that obese individuals have a lower proportion of Bacteroidetes, reduced bacterial diversity, and a higher proportion of Actinobacteria compared to their lean counterparts [243].

Further investigations have identified distinct microbial populations in obese individuals. For example, *Escherichia* and *Shigella* are amplified in the gut microbiota of obese patients, while families such as *Eubacterium coprostanoligenes*, Tannerellaceae, and other genera are more abundant in healthy individuals [244]. Additionally, specific microbiota biomarkers have been associated with various obesity classes, with Clostridiaceae serving as a marker for Class I obesity, *Bacilli* and *Lactobacillales* for Class II obesity, and certain pathogenic bacteria for Class III obesity [244]. Notably, *Enterobacter cloacae* isolated from morbidly obese individuals has been shown to induce both obesity and IR in germ‐free mice, with an increased abundance in morbidly obese individuals [245]. Bacteroides and Archaea also stand out in obese individuals, and their counts are relatively reduced in obese individuals undergoing treatment [246]. Studies on childhood obesity have also highlighted a correlation between elevated fecal concentrations of *Bacteroides fragilis* and *Lactobacillus* in obese children, which positively correlates with BMI, while the number of *Bifidobacterium* is higher in lean children and negatively correlated with BMI [247]. In addition, *Staphylococcus aureus* colonization has been associated with higher BMI [248]. Studies have shown that the number of *Escherichia coli* in obese women is higher than that in women with normal weight gain during pregnancy, while *Bifidobacterium* and *Akkermansia muciniphila* show the opposite trend [249]. In a double‐blind randomized controlled trial in obese adults, 12‐week synbiotic supplementation modestly reduced body weight and WC while increasing the abundance of beneficial genera such as *Bifidobacterium* and *Akkermansi*a [250].

The gut microbiome is also influenced by the OP and OR phenotypes observed in rodent models. In a study investigating these phenotypes under an HFD, male OP and OR mice exhibited distinct microbial abundances and metabolite profiles after 8 weeks, OP mice showed a decreased F/B ratio and an increase in Proteobacteria, in contrast to the microbial composition in OR and control mice [251]. A high‐calorie/fat diet has been shown to reduce gut microbiome diversity, diminishing the presence of beneficial bacteria that maintain intestinal barrier integrity, while promoting the proliferation of bacteria that compromise it [252, 253]. This diet also leads to dysbiosis and intestinal tissue disruption [254].

Moreover, the impact of obesity on the gut microbiome is not solely mediated by diet. In a study involving leptin‐deficient (Lep ob/ob) mice, which were fed the same diet as control mice for 16 weeks, significant changes in the gut microbiota were observed. Lep ob/ob mice showed an increase in Firmicutes, a reduction in Bacteroidetes, and an expansion of the Bacteroidetes order family_S24‐7 [255]. These findings underscore the complex nature of the gut microbiome's role in obesity, revealing that its characteristics cannot be simply categorized by increases or decreases in specific microbial populations [256].

### 7.3 Impact and Mechanism of Gut Flora on Obesity

The development of obesity is a complex pathological process that involves multiple factors, including imbalances in energy intake and expenditure, endocrine dysregulation, abnormal adipocyte function, and genetic predispositions. While previous studies have underscored the inextricable connection between obesity and factors such as the gut microbiome and diet, the precise underlying mechanisms and causal relationships remain unclear.

One study explored these mechanisms by introducing gut microbiota isolates from the fecal samples of 20 obese individuals into the diet of mice, and the results revealed that mice supplemented with the gut microbiota exhibited significantly elevated levels of total cholesterol, TAG, and low‐density lipoprotein, indicating that the altered microbiota of obese individuals could promote obesity development [257]. Further studies have shown that the gut microbiota in both obese mice and humans increases intestinal permeability and triggers chronic inflammation [258]. Mishra et al. proposed that reduced ethanolamine‐metabolizing bacteria in the gut of obese individuals lead to ethanolamine accumulation, which enhances ARID3a binding to the miR promoter, increasing miR‐101a‐103p expression. These miRNAs destabilize ZO‐1 mRNA, impairing the intestinal barrier, increasing permeability, and contributing to inflammation and dysregulated glucose metabolism [259].

Additionally, the metabolites produced by the gut microbiota can influence obesity through mechanisms such as DNA methylation. Dietary components significantly regulate the gut microbiota, which, in turn, affects the production of microbe‐derived metabolites that serve as substrates and enzyme regulators for epigenetic modifications, including DNA methylation, histone modifications, chromatin remodeling, and the regulation of gene expression by non‐coding RNAs [260]. A study focused on obesity induced by non‐high‐fat diets and demonstrated that obesity disrupts intestinal cell renewal; this disruption is characterized by increased expression of genes associated with cell death and survival/proliferation, as well as diminished expression of genes involved in tight junction formation and mucus synthesis, ultimately leading to increased intestinal permeability [255]. These changes in gut microbiota composition suggest that obesity primarily affects intestinal permeability, which may promote inflammation and endotoxemia even in the absence of a high‐fat diet. Recent studies have further revealed that inflammation induced by the gut microbiome can exacerbate obesity by increasing intestinal permeability and circulating levels of LPS, thereby contributing to the progression of obesity [261].

### 7.4 Possibility of Treating Obesity Through the Gut Microbiome

As mentioned above, the gut microbiome and obesity are closely interconnected, influencing and altering each other. Current obesity treatments primarily include nutritional interventions, physical activity, and pharmacotherapy [262]; however, these approaches have inherent limitations, such as suboptimal efficacy and numerous adverse effects [263].

Recent research into the gut microbiome has led to attempts at obesity treatment through the use of probiotics and other interventions. Miyamoto et al. [264] found that the gut microbiome can confer resistance to HFD‐induced obesity by producing polyunsaturated fatty acid (PUFA) metabolites, highlighting its protective role against HFD‐induced obesity. Additionally, other studies have identified bioactive metabolites in low‐concentration gut microbial PUFA metabolites, which may contribute to the development of functional foods for the prevention and treatment of obesity [265, 266].

A study exploring the impact of gut microbiota on intestinal permeability found that probiotic HL‐1200 therapy can reverse ethanolamine‐induced damage, including increased intestinal permeability, inflammation, and impaired glucose metabolism associated with T2D, while also reducing the elevated ethanolamine levels induced by the diet [259]. By investigating the effects and mechanisms of gut microbial populations on obesity, these findings offer valuable insights and potential avenues for developing more effective treatment strategies for obesity in the future.

Collectively, the evidence discussed above highlights that obesity is not driven by a single pathological factor, but rather by a highly integrated immunometabolic network. ATMs function as a central regulatory hub linking chronic inflammation, metabolic reprogramming, fatty acid dysregulation, IR, and gut microbiota alterations. These processes are tightly interconnected and mutually reinforcing, forming a self‐perpetuating cycle that drives the progression of obesity and its associated metabolic complications, and the key mechanisms, molecular mediators, and their functional consequences are summarized in Table 1.

**Table 1:** Immunometabolic dysregulation in obesity: Mechanisms and future perspectives.

Mechanistic module	Key cell/molecule	Change in obesity	Mechanisms	References
Macrophage polarization	M1/M2 macrophages	Shift from M2 to M1 phenotype	Promote chronic inflammation changes, exacerbate IR and metabolic dysfunction	[30, 57, 58, 61, 62, 63, 64, 65, 66, 67, 68, 69, 70, 71, 72, 73, 74]
Accumulation of macrophages	MCP1/CCL2,CCR2,MCP‐2/CCL8, MCP‐3/CCL7, MCP‐5/CCL12,IL‐20	Enhanced migration and local proliferation	Increase ATMs number, promote adipocyte hypertrophy and dysfunction	[20, 80, 81, 82, 83, 84, 85]
Efferocytosis	ATMs	Impaired clearance of dead adipocytes	Cause hyper‐inflammatory responses, chronic inflammation, and AT dysfunction	[20, 54, 86, 90, 91, 92]
Adipokines	Pro‐inflammatory adipokines (leptin, TNF, IL‐6, resistin, RBP4, LTP2, ANGPTL2, IL‐18, CCL2, CXCL5, NAMPT)/Anti‐inflammatory adipokines (lipocalin, SFRP5)	Pro‐inflammatory upregulation > anti‐inflammatory upregulation	Regulate monocyte recruitment, macrophage polarization, inflammation, and metabolic dysfunction	[93, 94, 95, 97]
Mitochondrial energy metabolism and metabolic reprogramming	M1/M2 macrophages, LPS, IFN‐γ	Glycolysis↑, FAO/OXPHOS↓, TCA cycle disrupted, promoting M1 polarization	Provide ATP, promote pro‐inflammatory cytokine secretion, chronic low‐grade inflammation, and metabolic dysregulation	[99, 100, 101, 102, 103, 104, 105, 106, 107, 108, 109, 110]
Insulin resistance	Leptin	Level increased	Impair insulin sensitivity, promote fat accumulation, and inflammation	[124, 125, 127]
	Adiponectin	Level decreased	Enhance insulin sensitivity, anti‐inflammatory	[5, 128]
	STEAP4	Downregulated in adipose tissue	Improves insulin sensitivity, exerts anti‐obesity effects	[132]
	Spexin/kisspeptin	Reduced in female obese patients	Negatively correlates with obesity and IR	[135]
	miR‐181b	Expression is reduced in AT endothelial cells	Impairs endothelial function and insulin sensitivity	[153]
	IRF5	Expression increased	Promote M1 polarization, reduce insulin sensitivity	[148, 149]
	IL‐29	Serum level increased	Reduce GLUT4 and AKT signaling, pro‐inflammatory	[147]
Fatty acid metabolism	Free fatty acids (FFA)	Release increased Ectopic deposition increased	Activate TLR4, induce oxidative stress, inhibit insulin signaling	[32, 56, 146]
	CD36	Overexpressed	Enhances fatty acid uptake, aggravates inflammation	[182, 184]
	ATGL/HSL	Expression levels are reduced in obese individuals	Impairs lipolysis, promotes lipid accumulation	[169, 170]
	FABP4	Upregulated	Induces macrophage apoptosis and lipotoxicity	[197]
	ACSL1/ACSL5	Overexpressed	Promotes lipid deposition, impairs insulin signaling	[202, 204]
Gut microbiota	Firmicutes/Bacteroidetes	Ratio is increased	Increases energy harvest, promotes obesity	[240, 241]
	*Bacteroides fragilis* and *Lactobacillus*	Abundance is elevated	Impairs gut barrier, enhances inflammation	[244, 249]
	*Bifidobacterium*	Abundance is decreased	Induces metabolic endotoxemia and systemic inflammation	[257, 258, 259, 261]

## 8 Conclusion and Prospect

Obesity is characterized by profound immunometabolic dysregulation in AT, in which macrophages act as central regulators linking chronic inflammation to metabolic dysfunction. In obese AT, macrophage infiltration is markedly increased and is associated with impaired lipogenesis and lipid storage, adipocyte necrosis, fibrosis, local inflammation, IR, and systemic metabolic disturbances [267]. Although macrophage activation may initially exert protective effects by clearing apoptotic adipocytes, removing excess lipids, and facilitating tissue repair, persistent nutrient excess and lipid overload drive sustained macrophage activation and metabolic reprogramming, ultimately promoting maladaptive inflammatory responses.

Here, we emphasize that obesity‐associated macrophage dysfunction cannot be fully explained by the traditional M1/M2 paradigm alone. Instead, we highlight the diversity of macrophage subsets with distinct inflammatory and metabolic properties, suggesting that these populations differentially regulate lipid handling, inflammatory signaling, tissue remodeling, and systemic metabolic homeostasis. This integrated approach offers a more comprehensive understanding of obesity‐related metabolic dysfunction and provides novel therapeutic targets for obesity and its associated metabolic disorders.

Furthermore, we underscore that fatty acid dysregulation and gut microbiota dysbiosis are deeply intertwined with macrophage‐mediated inflammation rather than representing independent pathological processes. Chronic elevation of FFAs disrupts glucose uptake and oxidation, inhibits glycogen synthesis, stimulates hepatic gluconeogenesis, and impairs insulin signaling and pancreatic β‐cell function [268]. Meanwhile, gut microbiota dysbiosis can alter host lipid metabolism, impair intestinal barrier integrity, enhance inflammatory signaling, and aggravate IR [269]. Accumulating evidence suggests a profound interplay among macrophage‐driven inflammation, metabolic dysfunction, and gut microbiota alterations in obesity. M1 macrophages exacerbate local AT inflammation, alter fatty acid metabolism, and facilitate lipid accumulation, thereby creating a maladaptive metabolic environment that extends to peripheral organs, including the liver, skeletal muscle, and pancreas [270]. Concurrently, gut microbiota dysbiosis amplifies inflammatory signaling and enhances macrophage polarization toward pro‐inflammatory phenotypes, thus perpetuating obesity‐related inflammation [271, 272]. Taken together, macrophage dysfunction, altered fatty acid metabolism, and gut microbiota dysbiosis should be viewed as interconnected components of a common pathogenic network rather than isolated drivers of obesity‐related disease.

In summary, this review provides an integrated overview of macrophage‐mediated inflammation, fatty acid metabolic disturbances, IR development, and gut microbiota alterations in obesity. By placing these processes within a macrophage‐centered immunometabolic framework, this article extends beyond prior topic‐specific discussions and offers a more comprehensive perspective on obesity‐associated metabolic dysfunction. Although significant progress has been made, further integrated research is still needed. Clarifying the precise mechanisms underlying macrophage phenotypic plasticity, fatty acid metabolism regulation, and microbiota‐host crosstalk will facilitate the development of innovative and targeted therapeutic strategies. Future studies leveraging multi‐omics and single‐cell technologies, together with other emerging approaches, may help establish more effective therapeutic paradigms for obesity‐induced metabolic diseases and ultimately improve clinical management and public health outcomes.

## Author Contributions

**Yuanyuan Li:** writing – original draft. **Jingwei Mao:** writing – original draft. **Yiwen Deng:** writing – review and editing. **Xiaobo Ge:** writing – review and editing. **Limin Nie:** visualization. **Qun Wang:** visualization. **Ruiqing Wang:** visualization. **Jiarun Zhang:** investigation. **Tianli Lyu:** investigation. **Luopeng Zhao:** conceptualization. **Lu Liu:** supervision. **Bin Li:** project administration.

## Conflicts of Interest

The authors declare no conflicts of interest.

## Data Availability

Data sharing is not applicable to this article as no datasets were generated or analyzed during the current study.

## References

[1] K. Chen , Z. Shen , W. Gu , et al., “Prevalence of Obesity and Associated Complications in China: A Cross‐Sectional, Real‐World Study in 15.8 Million Adults,” Diabetes, Obesity and Metabolism 25 (2023): 3390–3399. 10.1111/dom.15238 37589256

[2] W. T. Garvey , J. I. Mechanick , E. M. Brett , et al., “American Association of Clinical Endocrinologists and American College of Endocrinology Comprehensive Clinical Practice Guidelines for Medical Care of Patients With Obesity,” supplement, Endocrine Practice 22, no. Suppl 3 (2016): 1–203. 10.4158/EP161365.GL 27219496

[3] A. D. Hildreth , F. Ma , Y. Y. Wong , R. Sun , M. Pellegrini , and T. E. O'Sullivan , “Single‐Cell Sequencing of Human White Adipose Tissue Identifies New Cell States in Health and Obesity,” Nature Immunology 22 (2021): 639–653. 33907320 10.1038/s41590-021-00922-4 PMC8102391

[4] N. Perakakis , O. M. Farr , and C. S. Mantzoros , “Leptin in Leanness and Obesity,” Journal of the American College of Cardiology 77 (2021): 745–760. 33573745 10.1016/j.jacc.2020.11.069 PMC8483570

[5] T. Yamauchi and T. Kadowaki , “Adiponectin Receptor as a Key Player in Healthy Longevity and Obesity‐Related Diseases,” Cell Metabolism 17 (2013): 185–196. 23352188 10.1016/j.cmet.2013.01.001

[6] S. Sugimoto , H. A. Mena , B. E. Sansbury , et al., “Brown Adipose Tissue‐Derived MaR2 Contributes to Cold‐Induced Resolution of Inflammation,” Nature Metabolism 4 (2022): 775–790. 10.1038/s42255-022-00590-0 PMC9792164 35760872

[7] C. Cero , H. J. Lea , K. Y. Zhu , F. Shamsi , Y. H. Tseng , and A. M. Cypess , “β3‐Adrenergic Receptors Regulate Human Brown/Beige Adipocyte Lipolysis and Thermogenesis,” JCI Insight 6 (2021): e139160. 34100382 10.1172/jci.insight.139160 PMC8262278

[8] A. M. Cypess , L. S. Weiner , C. Roberts‐Toler , et al., “Activation of Human Brown Adipose Tissue by a β3‐Adrenergic Receptor Agonist,” Cell Metabolism 21 (2015): 33–38. 25565203 10.1016/j.cmet.2014.12.009 PMC4298351

[9] H. Shen , T. He , S. Wang , et al., “SOX4 Promotes Beige Adipocyte‐Mediated Adaptive Thermogenesis by Facilitating PRDM16‐PPARγ Complex,” Theranostics 12 (2022): 7699–7716. 36451857 10.7150/thno.77102 PMC9706582

[10] S. S. Choe , J. Y. Huh , I. J. Hwang , J. I. Kim , and J. B. Kim , “Adipose Tissue Remodeling: Its Role in Energy Metabolism and Metabolic Disorders,” Frontiers in Endocrinology 7 (2016): 30. 27148161 10.3389/fendo.2016.00030 PMC4829583

[11] B. Gustafson , S. Hedjazifar , S. Gogg , A. Hammarstedt , and U. Smith , “Insulin Resistance and Impaired Adipogenesis,” Trends in Endocrinology & Metabolism 26 (2015): 193–200. 25703677 10.1016/j.tem.2015.01.006

[12] A. Giordano , I. Murano , E. Mondini , et al., “Obese Adipocytes Show Ultrastructural Features of Stressed Cells and Die of Pyroptosis,” Journal of Lipid Research 54 (2013): 2423–2436. 23836106 10.1194/jlr.M038638 PMC3735940

[13] Y. S. Lee , J. Kim , O. Osborne , et al., “Increased Adipocyte O_2_ Consumption Triggers HIF‐1α, Causing Inflammation and Insulin Resistance in Obesity,” Cell 157 (2014): 1339–1352. 24906151 10.1016/j.cell.2014.05.012 PMC4114226

[14] S. Wueest , R. A. Rapold , J. M. Rytka , E. J. Schoenle , and D. Konrad , “Basal Lipolysis, Not the Degree of Insulin Resistance, Differentiates Large From Small Isolated Adipocytes in High‐Fat Fed Mice,” Diabetologia 52 (2009): 541–546. 19048227 10.1007/s00125-008-1223-5

[15] J. M. Rutkowski , J. H. Stern , and P. E. Scherer , “The Cell Biology of Fat Expansion,” Journal of Cell Biology 208 (2015): 501–512. 25733711 10.1083/jcb.201409063 PMC4347644

[16] J. I. Kim , J. Y. Huh , J. H. Sohn , et al., “Lipid‐Overloaded Enlarged Adipocytes Provoke Insulin Resistance Independent of Inflammation,” Molecular and Cellular Biology 35 (2015): 1686–1699. 25733684 10.1128/MCB.01321-14 PMC4405637

[17] T. V. Rohm , D. T. Meier , J. M. Olefsky , and M. Y. Donath , “Inflammation in Obesity, Diabetes, and Related Disorders,” Immunity 55 (2022): 31–55. 35021057 10.1016/j.immuni.2021.12.013 PMC8773457

[18] A. W. Ferrante, Jr. , “Obesity‐Induced Inflammation: A Metabolic Dialogue in the Language of Inflammation,” Journal of Internal Medicine 262 (2007): 408–414. 17875176 10.1111/j.1365-2796.2007.01852.x

[19] J. I. Odegaard and A. Chawla , “Alternative Macrophage Activation and Metabolism,” Annual Review of Pathology: Mechanisms of Disease 6 (2011): 275–297. 10.1146/annurev-pathol-011110-130138 PMC3381938 21034223

[20] S. P. Weisberg , D. McCann , M. Desai , M. Rosenbaum , R. L. Leibel , and A. W. Ferrante, Jr. , “Obesity Is Associated With Macrophage Accumulation in Adipose Tissue,” Journal of Clinical Investigation 112 (2003): 1796–1808. 14679176 10.1172/JCI19246 PMC296995

[21] H. Xu , G. T. Barnes , Q. Yang , et al., “Chronic Inflammation in Fat Plays a Crucial Role in the Development of Obesity‐Related Insulin Resistance,” Journal of Clinical Investigation 112 (2003): 1821–1830. 14679177 10.1172/JCI19451 PMC296998

[22] S. Guria , A. Hoory , S. Das , D. Chattopadhyay , and S. Mukherjee , “Adipose Tissue Macrophages and Their Role in Obesity‐Associated Insulin Resistance: An Overview of the Complex Dynamics at Play,” Bioscience Reports 43 (2023): BSR20220200, 10.1042/BSR20220200. 36718668 PMC10011338

[23] Y. Li , J. Hao , X. Kong , et al., “Rabeprazole Mitigates Obesity‐Induced Chronic Inflammation and Insulin Resistance Associated With Increased M2‐Type Macrophage Polarization,” Biochimica et Biophysica Acta (BBA) – Molecular Basis of Disease 1870 (2024): 167142. 38565384 10.1016/j.bbadis.2024.167142

[24] H. Zhao , Q. Shang , Z. Pan , et al., “Exosomes From Adipose‐Derived Stem Cells Attenuate Adipose Inflammation and Obesity Through Polarizing M2 Macrophages and Beiging in White Adipose Tissue,” Diabetes 67 (2018): 235–247. 29133512 10.2337/db17-0356

[25] Y. Li , S. Gao , S. Shi , et al., “Tetrahedral Framework Nucleic Acid‐Based Delivery of Resveratrol Alleviates Insulin Resistance: From Innate to Adaptive Immunity,” Nano‐Micro Letters 13 (2021): 86. 34138319 10.1007/s40820-021-00614-6 PMC8006527

[26] C. M. Stansbury , G. A. Dotson , H. Pugh , A. Rehemtulla , I. Rajapakse , and L. A. Muir , “A Lipid‐Associated Macrophage Lineage Rewires the Spatial Landscape of Adipose Tissue in Early Obesity,” JCI Insight 8 (2023): e171701. 37651193 10.1172/jci.insight.171701 PMC10619435

[27] X. Li , Y. Ren , K. Chang , et al., “Adipose Tissue Macrophages as Potential Targets for Obesity and Metabolic Diseases,” Frontiers in Immunology 14 (2023): 1153915. 37153549 10.3389/fimmu.2023.1153915 PMC10154623

[28] C. Choi , Y. L. Jeong , K. M. Park , et al., “TM4SF19‐Mediated Control of Lysosomal Activity in Macrophages Contributes to Obesity‐Induced Inflammation and Metabolic Dysfunction,” Nature Communications 15 (2024): 2779. 10.1038/s41467-024-47108-8 PMC10981689 38555350

[29] R. M. Pirzgalska , E. Seixas , J. S. Seidman , et al., “Sympathetic Neuron‐Associated Macrophages Contribute to Obesity by Importing and Metabolizing Norepinephrine,” Nature Medicine 23 (2017): 1309–1318. 10.1038/nm.4422 PMC7104364 29035364

[30] C. N. Lumeng , J. L. Bodzin , and A. R. Saltiel , “Obesity Induces a Phenotypic Switch in Adipose Tissue Macrophage Polarization,” Journal of Clinical Investigation 117 (2007): 175–184. 17200717 10.1172/JCI29881 PMC1716210

[31] T. Kawai , M. V. Autieri , and R. Scalia , “Adipose Tissue Inflammation and Metabolic Dysfunction in Obesity,” American Journal of Physiology‐Cell Physiology 320 (2021): C375–C391. 33356944 10.1152/ajpcell.00379.2020 PMC8294624

[32] C. Capurso and A. Capurso , “From Excess Adiposity to Insulin Resistance: The Role of Free Fatty Acids,” Vascular Pharmacology 57 (2012): 91–97. 22609131 10.1016/j.vph.2012.05.003

[33] P. Patel and N. Abate , “Body Fat Distribution and Insulin Resistance,” Nutrients 5 (2013): 2019–2027. 23739143 10.3390/nu5062019 PMC3725490

[34] A. Sica and A. Mantovani , “Macrophage Plasticity and Polarization: In Vivo Veritas,” Journal of Clinical Investigation 122 (2012): 787–795. 22378047 10.1172/JCI59643 PMC3287223

[35] S. K. Wculek , G. Dunphy , I. Heras‐Murillo , A. Mastrangelo , and D. Sancho , “Metabolism of Tissue Macrophages in Homeostasis and Pathology,” Cellular & Molecular Immunology 19 (2022): 384–408. 34876704 10.1038/s41423-021-00791-9 PMC8891297

[36] F. Ginhoux and M. Guilliams , “Tissue‐Resident Macrophage Ontogeny and Homeostasis,” Immunity 44 (2016): 439–449. 26982352 10.1016/j.immuni.2016.02.024

[37] C. F. Nathan , H. W. Murray , M. E. Wiebe , and B. Y. Rubin , “Identification of Interferon‐Gamma as the Lymphokine That Activates Human Macrophage Oxidative Metabolism and Antimicrobial Activity,” Journal of Experimental Medicine 158 (1983): 670–689. 6411853 10.1084/jem.158.3.670 PMC2187114

[38] M. Stein , S. Keshav , N. Harris , and S. Gordon , “Interleukin 4 Potently Enhances Murine Macrophage Mannose Receptor Activity: A Marker of Alternative Immunologic Macrophage Activation,” Journal of Experimental Medicine 176 (1992): 287–292. 1613462 10.1084/jem.176.1.287 PMC2119288

[39] C. D. Mills , K. Kincaid , J. M. Alt , M. J. Heilman , and A. M. Hill , “M‐1/M‐2 Macrophages and the Th1/Th2 Paradigm,” Journal of Immunology 164 (2000): 6166–6173. 10.4049/jimmunol.164.12.6166 10843666

[40] Q. Wang , H. Ni , L. Lan , X. Wei , R. Xiang , and Y. Wang , “Fra‐1 Protooncogene Regulates IL‐6 Expression in Macrophages and Promotes the Generation of M2d Macrophages,” Cell Research 20 (2010): 701–712. 20386569 10.1038/cr.2010.52

[41] J. Yao , D. Wu , and Y. Qiu , “Adipose Tissue Macrophage in Obesity‐Associated Metabolic Diseases,” Frontiers in Immunology 13 (2022): 977485. 36119080 10.3389/fimmu.2022.977485 PMC9478335

[42] M. Zeyda , K. Gollinger , E. Kriehuber , F. W. Kiefer , A. Neuhofer , and T. M. Stulnig , “Newly Identified Adipose Tissue Macrophage Populations in Obesity With Distinct Chemokine and Chemokine Receptor Expression,” International Journal of Obesity 34, no. 2005 (2010): 1684–1694. 20514049 10.1038/ijo.2010.103

[43] M. Kratz , B. R. Coats , K. B. Hisert , et al., “Metabolic Dysfunction Drives a Mechanistically Distinct Proinflammatory Phenotype in Adipose Tissue Macrophages,” Cell Metabolism 20 (2014): 614–625. 25242226 10.1016/j.cmet.2014.08.010 PMC4192131

[44] D. A. Hill , H. W. Lim , Y. H. Kim , et al., “Distinct Macrophage Populations Direct Inflammatory Versus Physiological Changes in Adipose Tissue,” Proceedings of the National Academy of Sciences 115 (2018): E5096–E5105. 10.1073/pnas.1802611115 PMC5984532 29760084

[45] D. A. Jaitin , L. Adlung , C. A. Thaiss , et al., “Lipid‐Associated Macrophages Control Metabolic Homeostasis in a Trem2‐Dependent Manner,” Cell 178 (2019): 686–698.e14. 31257031 10.1016/j.cell.2019.05.054 PMC7068689

[46] T. W. Benson , D. S. Weintraub , M. Crowe , et al., “Deletion of the Duffy Antigen Receptor for Chemokines (DARC) Promotes Insulin Resistance and Adipose Tissue Inflammation During High Fat Feeding,” Molecular and Cellular Endocrinology 473 (2018): 79–88. 29341885 10.1016/j.mce.2018.01.006 PMC6045443

[47] J. S. Orr , A. Kennedy , E. K. Anderson‐Baucum , et al., “Obesity Alters Adipose Tissue Macrophage Iron Content and Tissue Iron Distribution,” Diabetes 63 (2014): 421–432. 24130337 10.2337/db13-0213 PMC3900546

[48] J. H. Luo , F. X. Wang , J. W. Zhao , et al., “PDIA3 Defines a Novel Subset of Adipose Macrophages to Exacerbate the Development of Obesity and Metabolic Disorders,” Cell Metabolism 36 (2024): 2262–2280.e5. 39293433 10.1016/j.cmet.2024.08.009

[49] Y. N. Wang , Y. Tang , Z. He , et al., “Slit3 Secreted From M2‐Like Macrophages Increases Sympathetic Activity and Thermogenesis in Adipose Tissue,” Nature Metabolism 3 (2021): 1536–1551. 10.1038/s42255-021-00482-9 34782792

[50] L. C. Davies and P. R. Taylor , “Tissue‐Resident Macrophages: Then and Now,” Immunology 144 (2015): 541–548. 25684236 10.1111/imm.12451 PMC4368161

[51] N. Wang , H. Liang , and K. Zen , “Molecular Mechanisms That Influence the Macrophage m1‐m2 Polarization Balance,” Frontiers in Immunology 5 (2014): 614. 25506346 10.3389/fimmu.2014.00614 PMC4246889

[52] K. Sun , C. M. Kusminski , and P. E. Scherer , “Adipose Tissue Remodeling and Obesity,” Journal of Clinical Investigation 121 (2011): 2094–2101. 21633177 10.1172/JCI45887 PMC3104761

[53] A. Tajbakhsh , S. M. Gheibihayat , N. Karami , et al., “The Regulation of Efferocytosis Signaling Pathways and Adipose Tissue Homeostasis in Physiological Conditions and Obesity: Current Understanding and Treatment Options,” Obesity Reviews 23 (2022): e13487. 35765849 10.1111/obr.13487

[54] S. Cinti , G. Mitchell , G. Barbatelli , et al., “Adipocyte Death Defines Macrophage Localization and Function in Adipose Tissue of Obese Mice and Humans,” Journal of Lipid Research 46 (2005): 2347–2355. 16150820 10.1194/jlr.M500294-JLR200

[55] S. Russo , M. Kwiatkowski , N. Govorukhina , R. Bischoff , and B. N. Melgert , “Meta‐Inflammation and Metabolic Reprogramming of Macrophages in Diabetes and Obesity: The Importance of Metabolites,” Frontiers in Immunology 12 (2021): 746151. 34804028 10.3389/fimmu.2021.746151 PMC8602812

[56] H. Shi , M. V. Kokoeva , K. Inouye , I. Tzameli , H. Yin , and J. S. Flier , “TLR4 Links Innate Immunity and Fatty Acid‐Induced Insulin Resistance,” Journal of Clinical Investigation 116 (2006): 3015–3025. 17053832 10.1172/JCI28898 PMC1616196

[57] A. Shapouri‐Moghaddam , S. Mohammadian , H. Vazini , et al., “Macrophage Plasticity, Polarization, and Function in Health and Disease,” Journal of Cellular Physiology 233 (2018): 6425–6440. 10.1002/jcp.26429 PMC13482560 29319160

[58] A. Mantovani , A. Sica , S. Sozzani , P. Allavena , A. Vecchi , and M. Locati , “The Chemokine System in Diverse Forms of Macrophage Activation and Polarization,” Trends in Immunology 25 (2004): 677–686. 15530839 10.1016/j.it.2004.09.015

[59] S. K. Biswas , M. Chittezhath , I. N. Shalova , and J. Y. Lim , “Macrophage Polarization and Plasticity in Health and Disease,” Immunologic Research 53 (2012): 11–24. 22418728 10.1007/s12026-012-8291-9

[60] P. J. Murray , J. E. Allen , S. K. Biswas , et al., “Macrophage Activation and Polarization: Nomenclature and Experimental Guidelines,” Immunity 41 (2014): 14–20. 25035950 10.1016/j.immuni.2014.06.008 PMC4123412

[61] P. S. Lee , C. Y. Teng , K. F. Hsieh , et al., “Adzuki Bean Water Extract Attenuates Obesity by Modulating M2/M1 Macrophage Polarization and Gut Microbiota Composition,” Molecular Nutrition & Food Research 63 (2019): e1900626. 31574574 10.1002/mnfr.201900626

[62] K. Essandoh , Y. Li , J. Huo , and G. C. Fan , “MiRNA‐Mediated Macrophage Polarization and Its Potential Role in the Regulation of Inflammatory Response,” Shock 46 (2016): 122–131. 26954942 10.1097/SHK.0000000000000604 PMC4949115

[63] L. Chávez‐Galán , M. L. Olleros , D. Vesin , and I. Garcia , “Much More Than M1 and M2 Macrophages, There Are Also CD169(+) and TCR(+) Macrophages,” Frontiers in Immunology 6 (2015): 263. 26074923 10.3389/fimmu.2015.00263 PMC4443739

[64] L. G. Bekker , S. Freeman , P. J. Murray , B. Ryffel , and G. Kaplan , “TNF‐α Controls Intracellular Mycobacterial Growth by Both Inducible Nitric Oxide Synthase‐Dependent and Inducible Nitric Oxide Synthase‐Independent Pathways,” Journal of Immunology 166 (2001): 6728–6734. 10.4049/jimmunol.166.11.6728 11359829

[65] S. Ehlers , S. Kutsch , E. M. Ehlers , J. Benini , and K. Pfeffer , “Lethal Granuloma Disintegration in Mycobacteria‐Infected TNFRp55−/− Mice Is Dependent on T Cells and IL‐12,” Journal of Immunology 165 (2000): 483–492. 10.4049/jimmunol.165.1.483 10861087

[66] J. Braune , U. Weyer , C. Hobusch , et al., “IL‐6 Regulates M2 Polarization and Local Proliferation of Adipose Tissue Macrophages in Obesity,” Journal of Immunology 198 (2017): 2927–2934. 10.4049/jimmunol.1600476 28193830

[67] A. A. Tarique , J. Logan , E. Thomas , P. G. Holt , P. D. Sly , and E. Fantino , “Phenotypic, Functional, and Plasticity Features of Classical and Alternatively Activated Human Macrophages,” American Journal of Respiratory Cell and Molecular Biology 53 (2015): 676–688. 25870903 10.1165/rcmb.2015-0012OC

[68] C. Atri , F. Z. Guerfali , and D. Laouini , “Role of Human Macrophage Polarization in Inflammation During Infectious Diseases,” International Journal of Molecular Sciences 19 (2018): 1801. 29921749 10.3390/ijms19061801 PMC6032107

[69] S. Gordon , “Alternative Activation of Macrophages,” Nature Reviews Immunology 3 (2003): 23–35. 10.1038/nri978 12511873

[70] S. Gordon and P. R. Taylor , “Monocyte and Macrophage Heterogeneity,” Nature Reviews Immunology 5 (2005): 953–964. 10.1038/nri1733 16322748

[71] Y. H. Hsu , C. H. Wu , C. J. Chiu , et al., “IL‐20 Is Involved in Obesity by Modulation of Adipogenesis and Macrophage Dysregulation,” Immunology 164 (2021): 817–833. 34403503 10.1111/imm.13403 PMC8561105

[72] S. Gordon and F. O. Martinez , “Alternative Activation of Macrophages: Mechanism and Functions,” Immunity 32 (2010): 593–604. 20510870 10.1016/j.immuni.2010.05.007

[73] Y. C. Liu , X. B. Zou , Y. F. Chai , and Y. M. Yao , “Macrophage Polarization in Inflammatory Diseases,” International Journal of Biological Sciences 10 (2014): 520–529. 24910531 10.7150/ijbs.8879 PMC4046879

[74] C. N. Lumeng , J. B. DelProposto , D. J. Westcott , and A. R. Saltiel , “Phenotypic Switching of Adipose Tissue Macrophages With Obesity Is Generated by Spatiotemporal Differences in Macrophage Subtypes,” Diabetes 57 (2008): 3239–3246. 18829989 10.2337/db08-0872 PMC2584129

[75] S. E. Gambaro , M. G. Zubiría , A. E. Portales , M. A. Rey , M. Rumbo , and A. Giovambattista , “M1 Macrophage Subtypes Activation and Adipocyte Dysfunction Worsen During Prolonged Consumption of a Fructose‐Rich Diet,” Journal of Nutritional Biochemistry 61 (2018): 173–182. 30245336 10.1016/j.jnutbio.2018.08.004

[76] W. Luo , L. Ai , B. Wang , et al., “Eccentric Exercise and Dietary Restriction Inhibits M1 Macrophage Polarization Activated by High‐Fat Diet‐Induced Obesity,” Life Sciences 243 (2020): 117246. 31904367 10.1016/j.lfs.2019.117246

[77] J. H. Lee , S. H. Lee , E. H. Lee , et al., “SCAP Deficiency Facilitates Obesity and Insulin Resistance Through Shifting Adipose Tissue Macrophage Polarization,” Journal of Advanced Research 45 (2023): 1–13. 35659922 10.1016/j.jare.2022.05.013 PMC10006517

[78] X. Zhang , J. Li , S. Luo , et al., “IgE Contributes to Atherosclerosis and Obesity by Affecting Macrophage Polarization, Macrophage Protein Network, and Foam Cell Formation,” Arteriosclerosis, Thrombosis, and Vascular Biology 40 (2020): 597–610. 31996021 10.1161/ATVBAHA.119.313744 PMC7047522

[79] F. Qin , W. Huang , C. Qu , et al., “The Effects of Exercise on MicroRNA Expression Profiling in Adipose Tissue Macrophages of Mice,” Frontiers in Immunology 15 (2024): 1412621. 39224599 10.3389/fimmu.2024.1412621 PMC11366585

[80] Y. Wu , C. Wu , T. Shi , et al., “FAP Expression in Adipose Tissue Macrophages Promotes Obesity and Metabolic Inflammation,” Proceedings of the National Academy of Sciences 120 (2023): e2303075120. 10.1073/pnas.2303075120 PMC10743525 38100414

[81] L. Russo and C. N. Lumeng , “Properties and Functions of Adipose Tissue Macrophages in Obesity,” Immunology 155 (2018): 407–417. 30229891 10.1111/imm.13002 PMC6230999

[82] J. C. McNelis and J. M. Olefsky , “Macrophages, Immunity, and Metabolic Disease,” Immunity 41 (2014): 36–48. 25035952 10.1016/j.immuni.2014.05.010

[83] H. Kanda , “MCP‐1 Contributes to Macrophage Infiltration into Adipose Tissue, Insulin Resistance, and Hepatic Steatosis in Obesity,” Journal of Clinical Investigation 116 (2006): 1494–1505. 16691291 10.1172/JCI26498 PMC1459069

[84] S. P. Weisberg , D. Hunter , R. Huber , et al., “CCR2 Modulates Inflammatory and Metabolic Effects of High‐Fat Feeding,” Journal of Clinical Investigation 116 (2006): 115–124. 16341265 10.1172/JCI24335 PMC1307559

[85] C. Zheng , Q. Yang , J. Cao , et al., “Local Proliferation Initiates Macrophage Accumulation in Adipose Tissue During Obesity,” Cell Death & Disease 7 (2016): e2167. 27031964 10.1038/cddis.2016.54 PMC4823955

[86] Y. Tao , J. Zang , T. Wang , et al., “Obesity‐Associated Macrophages Dictate Adipose Stem Cell Ferroptosis and Visceral Fat Dysfunction by Propagating Mitochondrial Fragmentation,” Nature Communications 16 (2025): 7564. 10.1038/s41467-025-62690-1 PMC12354832 40813577

[87] M. Tardelli , K. Zeyda , V. Moreno‐Viedma , et al., “Osteopontin Is a Key Player for Local Adipose Tissue Macrophage Proliferation in Obesity,” Molecular Metabolism 5 (2016): 1131–1137. 27818939 10.1016/j.molmet.2016.09.003 PMC5081407

[88] W. Ying , Y. S. Lee , Y. Dong , et al., “Expansion of Islet‐Resident Macrophages Leads to Inflammation Affecting β Cell Proliferation and Function in Obesity,” Cell Metabolism 29 (2019): 457–474.e5. 30595478 10.1016/j.cmet.2018.12.003 PMC6701710

[89] L. Wang , Y. Wang , C. Zhang , et al., “Inhibiting Glycogen Synthase Kinase 3 Reverses Obesity‐Induced White Adipose Tissue Inflammation by Regulating Apoptosis Inhibitor of Macrophage/CD5L‐Mediated Macrophage Migration,” Arteriosclerosis, Thrombosis, and Vascular Biology 38 (2018): 2103–2116. 30026270 10.1161/ATVBAHA.118.311363

[90] T. McLaughlin , S. E. Ackerman , L. Shen , and E. Engleman , “Role of Innate and Adaptive Immunity in Obesity‐Associated Metabolic Disease,” Journal of Clinical Investigation 127 (2017): 5–13. 28045397 10.1172/JCI88876 PMC5199693

[91] A. Lindhorst , N. Raulien , P. Wieghofer , et al., “Adipocyte Death Triggers a Pro‐Inflammatory Response and Induces Metabolic Activation of Resident Macrophages,” Cell Death & Disease 12 (2021): 579. 34091595 10.1038/s41419-021-03872-9 PMC8179930

[92] A. C. Doran , A. Yurdagul, Jr. , and I. Tabas , “Efferocytosis in Health and Disease,” Nature Reviews Immunology 20 (2020): 254–267. 10.1038/s41577-019-0240-6 PMC7667664 31822793

[93] X. Unamuno , J. Gómez‐Ambrosi , A. Rodríguez , S. Becerril , G. Frühbeck , and V. Catalán , “Adipokine Dysregulation and Adipose Tissue Inflammation in Human Obesity,” European Journal of Clinical Investigation 48 (2018): e12997. 29995306 10.1111/eci.12997

[94] N. Ouchi , J. L. Parker , J. J. Lugus , and K. Walsh , “Adipokines in Inflammation and Metabolic Disease,” Nature Reviews Immunology 11 (2011): 85–97. 10.1038/nri2921 PMC3518031 21252989

[95] V. Murahovschi , O. Pivovarova , I. Ilkavets , et al., “WISP1 Is a Novel Adipokine Linked to Inflammation in Obesity,” Diabetes 64 (2015): 856–866. 25281430 10.2337/db14-0444

[96] S. He , J. Ryu , J. Liu , et al., “LRG1 Is an Adipokine That Mediates Obesity‐Induced Hepatosteatosis and Insulin Resistance,” Journal of Clinical Investigation 131 (2021): e148545. 34730111 10.1172/JCI148545 PMC8670837

[97] B. Li , S. Sun , J. J. Li , J. P. Yuan , S. R. Sun , and Q. Wu , “Adipose Tissue Macrophages: Implications for Obesity‐Associated Cancer,” Military Medical Research 10, no. 1 (2023): 1. 36593475 10.1186/s40779-022-00437-5 PMC9809128

[98] K. H. E. Chen , N. M. Lainez , and D. Coss , “Sex Differences in Macrophage Responses to Obesity‐Mediated Changes Determine Migratory and Inflammatory Traits,” Journal of Immunology 206 (2021): 141–153. 10.4049/jimmunol.2000490 PMC8903060 33268480

[99] S. Heinonen , R. Jokinen , A. Rissanen , and K. H. Pietiläinen , “White Adipose Tissue Mitochondrial Metabolism in Health and in Obesity,” Obesity Reviews 21 (2020): e12958. 31777187 10.1111/obr.12958

[100] J. Feng , L. Li , Z. Ou , et al., “IL‐25 Stimulates M2 Macrophage Polarization and Thereby Promotes Mitochondrial Respiratory Capacity and Lipolysis in Adipose Tissues Against Obesity,” Cellular & Molecular Immunology 15 (2018): 493–505. 28194019 10.1038/cmi.2016.71 PMC6068125

[101] L. Orliaguet , T. Ejlalmanesh , A. Humbert , et al., “Early Macrophage Response to Obesity Encompasses Interferon Regulatory Factor 5 Regulated Mitochondrial Architecture Remodelling,” Nature Communications 13 (2022): 5089. 10.1038/s41467-022-32813-z PMC9427774 36042203

[102] A. Viola , F. Munari , R. Sánchez‐Rodríguez , T. Scolaro , and A. Castegna , “The Metabolic Signature of Macrophage Responses,” Frontiers in Immunology 10 (2019): 1462. 31333642 10.3389/fimmu.2019.01462 PMC6618143

[103] Y. Liu , R. Xu , H. Gu , et al., “Metabolic Reprogramming in Macrophage Responses,” Biomarker Research 9, no. 1 (2021): 1. 33407885 10.1186/s40364-020-00251-y PMC7786975

[104] L. Boutens , G. J. Hooiveld , S. Dhingra , R. A. Cramer , M. G. Netea , and R. Stienstra , “Unique Metabolic Activation of Adipose Tissue Macrophages in Obesity Promotes Inflammatory Responses,” Diabetologia 61 (2018): 942–953. 29333574 10.1007/s00125-017-4526-6 PMC6448980

[105] S. E. Corcoran and L. A. J. O'Neill , “HIF1α and Metabolic Reprogramming in Inflammation,” Journal of Clinical Investigation 126 (2016): 3699–3707. 27571407 10.1172/JCI84431 PMC5096812

[106] D. G. Ryan and L. A. J. O'Neill , “Krebs Cycle Reborn in Macrophage Immunometabolism,” Annual Review of Immunology 38 (2020): 289–313. 10.1146/annurev-immunol-081619-104850 31986069

[107] E. Rendra , V. Riabov , D. M. Mossel , T. Sevastyanova , M. C. Harmsen , and J. Kzhyshkowska , “Reactive Oxygen Species (ROS) in Macrophage Activation and Function in Diabetes,” Immunobiology 224 (2019): 242–253. 30739804 10.1016/j.imbio.2018.11.010

[108] Y. H. Kang , M. H. Cho , J. Y. Kim , et al., “Impaired Macrophage Autophagy Induces Systemic Insulin Resistance in Obesity,” Oncotarget 7 (2016): 35577–35591. 27229537 10.18632/oncotarget.9590 PMC5094946

[109] P. Petrus , S. Lecoutre , L. Dollet , et al., “Glutamine Links Obesity to Inflammation in Human White Adipose Tissue,” Cell Metabolism 31 (2020): 375–390.e11. 31866443 10.1016/j.cmet.2019.11.019

[110] J. E. Bader , M. M. Wolf , G. L. Lupica‐Tondo , et al., “Obesity Induces PD‐1 on Macrophages to Suppress Anti‐Tumour Immunity,” Nature 630 (2024): 968–975. 38867043 10.1038/s41586-024-07529-3 PMC11456854

[111] E. K. Sims , A. L. J. Carr , R. A. Oram , L. A. DiMeglio , and C. Evans‐Molina , “100 Years of Insulin: Celebrating the Past, Present and Future of Diabetes Therapy,” Nature Medicine 27 (2021): 1154–1164. 10.1038/s41591-021-01418-2 PMC8802620 34267380

[112] S. Polsky and S. L. Ellis , “Obesity, Insulin Resistance, and Type 1 Diabetes Mellitus,” Current Opinion in Endocrinology, Diabetes & Obesity 22 (2015): 277–282. 26087341 10.1097/MED.0000000000000170

[113] E. Orgel and S. D. Mittelman , “The Links Between Insulin Resistance, Diabetes, and Cancer,” Current Diabetes Reports 13 (2013): 213–222. 23271574 10.1007/s11892-012-0356-6 PMC3595327

[114] A. O. Gobato , A. C. J. Vasques , M. P. Zambon , A. A. A. Barros Filho , and G. Hessel , “Metabolic Syndrome and Insulin Resistance in Obese Adolescents,” Revista Paulista de Pediatria 32 (2014): 55–59. 24676191 10.1590/S0103-05822014000100010 PMC4182990

[115] A. M. Sharma and V. T. Chetty , “Obesity, Hypertension and Insulin Resistance,” Acta Diabetologica 42, no. Suppl 1 (2005): s3–s8. 15868117 10.1007/s00592-005-0175-1

[116] B. Ahmed , R. Sultana , and M. W. Greene , “Adipose Tissue and Insulin Resistance in Obese,” Biomedicine & Pharmacotherapy 137 (2021): 111315. 33561645 10.1016/j.biopha.2021.111315

[117] S. N. F. Assunção , N. C. A. Boa Sorte , C. A. D. Alves , P. S. A. Mendes , C. R. B. Alves , and L. R. Silva , “Glucose Alteration and Insulin Resistance in Asymptomatic Obese Children and Adolescents,” Jornal de Pediatria 94 (2018): 268–272. 28850814 10.1016/j.jped.2017.06.008

[118] R. A. Al‐Horani , K. M. Alsays , and O. Abo Alrob , “Obesity Blunts Insulin Sensitivity Improvements and Attenuates Strength Gains Following Resistance Training in Nondiabetic Men,” European Journal of Applied Physiology 124 (2024): 1425–1437. 38100040 10.1007/s00421-023-05370-6

[119] D. E. James , J. Stöckli , and M. J. Birnbaum , “The Aetiology and Molecular Landscape of Insulin Resistance,” Nature Reviews Molecular Cell Biology 22 (2021): 751–771. 34285405 10.1038/s41580-021-00390-6

[120] I. Nieto‐Vazquez , S. Fernández‐Veledo , D. K. Krämer , R. Vila‐Bedmar , L. Garcia‐Guerra , and M. Lorenzo , “Insulin Resistance Associated to Obesity: The Link TNF‐Alpha,” Archives of Physiology and Biochemistry 114 (2008): 183–194. 18629684 10.1080/13813450802181047

[121] Z. Qing , W. Xiao‐Hui , W. Xi‐Mei , and Z. Chao‐Chun , “Vitamin C Deficiency Aggravates Tumor Necrosis Factor α‐Induced Insulin Resistance,” European Journal of Pharmacology 829 (2018): 1–11. 29625084 10.1016/j.ejphar.2018.03.044

[122] N. K. Maclaren , S. Gujral , S. Ten , and R. Motagheti , “Childhood Obesity and Insulin Resistance,” Cell Biochemistry and Biophysics 48 (2007): 73–78. 10.1007/s12013-007-0017-6 17709876

[123] C. Maffeis , P. Moghetti , A. Grezzani , M. Clementi , R. Gaudino , and L. Tatò , “Insulin Resistance and the Persistence of Obesity From Childhood Into Adulthood,” Journal of Clinical Endocrinology & Metabolism 87 (2002): 71–76. 11788625 10.1210/jcem.87.1.8130

[124] J. F. Davis , D. L. Choi , and S. C. Benoit , “Insulin, Leptin and Reward,” Trends in Endocrinology & Metabolism 21 (2010): 68–74. 19818643 10.1016/j.tem.2009.08.004 PMC2822063

[125] T. J. Kieffer , R. S. Heller , C. A. Leech , G. G. Holz , and J. F. Habener , “Leptin Suppression of Insulin Secretion by the Activation of ATP‐Sensitive K+ Channels in Pancreatic β‐Cells,” Diabetes 46 (1997): 1087–1093. 9166685 10.2337/diab.46.6.1087 PMC2940064

[126] V. Emilsson , Y. L. Liu , M. A. Cawthorne , N. M. Morton , and M. Davenport , “Expression of the Functional Leptin Receptor mRNA in Pancreatic Islets and Direct Inhibitory Action of Leptin on Insulin Secretion,” Diabetes 46 (1997): 313–316. 9000710 10.2337/diab.46.2.313

[127] H. Laivuori , R. Kaaja , H. Koistinen , et al., “Leptin During and After Preeclamptic or Normal Pregnancy: Its Relation to Serum Insulin and Insulin Sensitivity,” Metabolism: Clinical and Experimental 49 (2000): 259–263. 10690955 10.1016/s0026-0495(00)91559-2

[128] A. Engin , “Adiponectin‐Resistance in Obesity,” Advances in Experimental Medicine and Biology 960 (2017): 415–441. 28585210 10.1007/978-3-319-48382-5_18

[129] N. Celik , N. Andiran , and A. E. Yilmaz , “The Relationship Between Serum Magnesium Levels With Childhood Obesity and Insulin Resistance: A Review of the Literature,” Journal of Pediatric Endocrinology & Metabolism: JPEM 24 (2011): 675–678. 22145455

[130] E. Isganaitis and R. H. Lustig , “Fast Food, Central Nervous System Insulin Resistance, and Obesity,” Arteriosclerosis, Thrombosis, and Vascular Biology 25 (2005): 2451–2462. 16166564 10.1161/01.ATV.0000186208.06964.91

[131] J. Jiang , X. Cai , Y. Pan , et al., “Relationship of Obesity to Adipose Tissue Insulin Resistance,” BMJ Open Diabetes Research & Care 8 (2020): e000741. 10.1136/bmjdrc-2019-000741 PMC7254100 32245824

[132] X. Chen , Z. Huang , B. Zhou , et al., “STEAP4 and Insulin Resistance,” Endocrine 47 (2014): 372–379. 24627165 10.1007/s12020-014-0230-1

[133] S. Karakaya , M. Altay , F. Kaplan Efe , et al., “The Neutrophil‐Lymphocyte Ratio and Its Relationship With Insulin Resistance in Obesity,” Turkish Journal of Medical Sciences 49 (2019): 245–248. 30761879 10.3906/sag-1804-68 PMC7350826

[134] R. Yang , Y. Hu , C. H. Lee , et al., “PM20D1 Is a Circulating Biomarker Closely Associated With Obesity, Insulin Resistance and Metabolic Syndrome,” European Journal of Endocrinology 186 (2022): 151–161. 10.1530/EJE-21-0847 34757919

[135] P. A. Kołodziejski , E. Pruszyńska‐Oszmałek , E. Korek , et al., “Serum Levels of Spexin and Kisspeptin Negatively Correlate With Obesity and Insulin Resistance in Women,” Physiological Research 67 (2018): 45–56. 29137471 10.33549/physiolres.933467

[136] O. Akın , I. Eker , M. Arslan , et al., “Association of Nerve Conduction Impairment and Insulin Resistance in Children With Obesity,” Child's Nervous System 32 (2016): 2219–2224. 10.1007/s00381-016-3210-3 27503137

[137] A. Prats‐Puig , F. J. Ortega , J. M. Mercader , et al., “Changes in Circulating MicroRNAs Are Associated With Childhood Obesity,” Journal of Clinical Endocrinology & Metabolism 98 (2013): E1655–E1660. 23928666 10.1210/jc.2013-1496

[138] R. Wang , J. Hong , Y. Cao , et al., “Elevated Circulating MicroRNA‐122 Is Associated With Obesity and Insulin Resistance in Young Adults,” European Journal of Endocrinology 172 (2015): 291–300. 25515554 10.1530/EJE-14-0867

[139] P. A. Sarafidis , “Obesity, Insulin Resistance and Kidney Disease Risk: Insights Into the Relationship,” Current Opinion in Nephrology and Hypertension 17 (2008): 450–456. 18695384 10.1097/MNH.0b013e328305b994

[140] J. C. Frisbee , “Obesity, Insulin Resistance, and Microvessel Density,” Microcirculation 14 (2007): 289–298. 17613802 10.1080/10739680701282945

[141] F. Armutcu and E. McCloskey , “Insulin Resistance, Bone Health, and Fracture Risk,” Osteoporosis International 35 (2024): 1909–1917. 39264439 10.1007/s00198-024-07227-w

[142] H. Li , Y. Meng , S. He , et al., “Macrophages, Chronic Inflammation, and Insulin Resistance,” Cells 11 (2022): 3001. 36230963 10.3390/cells11193001 PMC9562180

[143] C. N. Lumeng , S. M. Deyoung , and A. R. Saltiel , “Macrophages Block Insulin Action in Adipocytes by Altering Expression of Signaling and Glucose Transport Proteins,” American Journal of Physiology‐Endocrinology and Metabolism 292 (2007): E166–E174. 16926380 10.1152/ajpendo.00284.2006 PMC3888778

[144] S. Tateya , F. Kim , and Y. Tamori , “Recent Advances in Obesity‐Induced Inflammation and Insulin Resistance,” Frontiers in Endocrinology 4 (2013): 93. 23964268 10.3389/fendo.2013.00093 PMC3737462

[145] K. Kang , S. M. Reilly , V. Karabacak , et al., “Adipocyte‐Derived Th2 Cytokines and Myeloid PPARδ Regulate Macrophage Polarization and Insulin Sensitivity,” Cell Metabolism 7 (2008): 485–495. 18522830 10.1016/j.cmet.2008.04.002 PMC2586840

[146] G. R. Steinberg , “Inflammation in Obesity Is a Common Link Between Defects in Fatty Acid Metabolism and Insulin Resistance,” Cell Cycle 6 (2007): 888–894. 17438370 10.4161/cc.6.8.4135

[147] T. Y. Lin , C. J. Chiu , C. H. Kuan , et al., “IL‐29 Promoted Obesity‐Induced Inflammation and Insulin Resistance,” Cellular & Molecular Immunology 17 (2020): 369–379. 31363171 10.1038/s41423-019-0262-9 PMC7109060

[148] T. Krausgruber , K. Blazek , T. Smallie , et al., “IRF5 Promotes Inflammatory Macrophage Polarization and TH1‐TH17 Responses,” Nature Immunology 12 (2011): 231–238. 21240265 10.1038/ni.1990

[149] E. Dalmas , A. Toubal , F. Alzaid , et al., “Irf5 Deficiency in Macrophages Promotes Beneficial Adipose Tissue Expansion and Insulin Sensitivity During Obesity,” Nature Medicine 21 (2015): 610–618. 10.1038/nm.3829 25939064

[150] Y. Zhu , J. Tian , X. Wei , S. Jia , and Q. Shu , “Electroacupuncture Alleviates Obesity and Insulin Resistance via the GLP‐1‐VTA^DA^ Reward Circuit,” Neuroendocrinology 114 (2024): 263–278. 37989106 10.1159/000535068

[151] S. Shaw and P. Boden , “Insulin Resistance, Obesity and the Metabolic Syndrome. Is There a Therapeutic Role for Endothelin‐1 Antagonists?,” Current Vascular Pharmacology 3 (2005): 359–363. 16248779 10.2174/157016105774329471

[152] J. Wittwer and D. Bradley , “Clusterin and Its Role in Insulin Resistance and the Cardiometabolic Syndrome,” Frontiers in Immunology 12 (2021): 612496. 33717095 10.3389/fimmu.2021.612496 PMC7946829

[153] X. Sun , J. Lin , Y. Zhang , et al., “MicroRNA‐181b Improves Glucose Homeostasis and Insulin Sensitivity by Regulating Endothelial Function in White Adipose Tissue,” Circulation Research 118 (2016): 810–821. 26830849 10.1161/CIRCRESAHA.115.308166 PMC4779381

[154] R. K. Zwick , C. F. Guerrero‐Juarez , V. Horsley , and M. V. Plikus , “Anatomical, Physiological, and Functional Diversity of Adipose Tissue,” Cell Metabolism 27 (2018): 68–83. 29320711 10.1016/j.cmet.2017.12.002 PMC6050204

[155] J. A. Villena , B. Viollet , F. Andreelli , A. Kahn , S. Vaulont , and H. S. Sul , “Induced Adiposity and Adipocyte Hypertrophy in Mice Lacking the AMP‐Activated Protein Kinase‐α2 Subunit,” Diabetes 53, no. 9 (2004): 2242–2249. 15331533 10.2337/diabetes.53.9.2242

[156] T. O. Eichmann , M. Kumari , J. T. Haas , et al., “Studies on the Substrate and Stereo/Regioselectivity of Adipose Triglyceride Lipase, Hormone‐Sensitive Lipase, and Diacylglycerol‐O‐Acyltransferases,” Journal of Biological Chemistry 287 (2012): 41446–41457. 23066022 10.1074/jbc.M112.400416 PMC3510842

[157] R. Zimmermann , J. G. Strauss , G. Haemmerle , et al., “Fat Mobilization in Adipose Tissue Is Promoted by Adipose Triglyceride Lipase,” Science 306 (2004): 1383–1386. 15550674 10.1126/science.1100747

[158] E. E. Kershaw , J. K. Hamm , L. A. W. Verhagen , O. Peroni , M. Katic , and J. S. Flier , “Adipose Triglyceride Lipase,” Diabetes 55 (2006): 148–157. 16380488 PMC2819178

[159] J. A. Villena , S. Roy , E. Sarkadi‐Nagy , K. H. Kim , and H. S. Sul , “Desnutrin, an Adipocyte Gene Encoding a Novel Patatin Domain‐Containing Protein, Is Induced by Fasting and Glucocorticoids,” Journal of Biological Chemistry 279 (2004): 47066–47075. 15337759 10.1074/jbc.M403855200

[160] J. G. Granneman , H. P. H. Moore , R. Krishnamoorthy , and M. Rathod , “Perilipin Controls Lipolysis by Regulating the Interactions of AB‐Hydrolase Containing 5 (Abhd5) and Adipose Triglyceride Lipase (Atgl),” Journal of Biological Chemistry 284 (2009): 34538–34544. 19850935 10.1074/jbc.M109.068478 PMC2787315

[161] S. Kaushik and A. M. Cuervo , “AMPK‐Dependent Phosphorylation of Lipid Droplet Protein PLIN2 Triggers Its Degradation by CMA,” Autophagy 12 (2016): 432–438. 26902588 10.1080/15548627.2015.1124226 PMC4835968

[162] X. Yang , B. L. Heckmann , X. Zhang , C. M. Smas , and J. Liu , “Distinct Mechanisms Regulate ATGL‐Mediated Adipocyte Lipolysis by Lipid Droplet Coat Proteins,” Molecular Endocrinology 27 (2013): 116–126. 23204327 10.1210/me.2012-1178 PMC3545209

[163] A. Lass , R. Zimmermann , G. Haemmerle , et al., “Adipose Triglyceride Lipase‐Mediated Lipolysis of Cellular Fat Stores Is Activated by CGI‐58 and Defective in Chanarin‐Dorfman Syndrome,” Cell Metabolism 3 (2006): 309–319. 16679289 10.1016/j.cmet.2006.03.005

[164] X. Yang , X. Lu , M. Lombès , et al., “The G(0)/G(1) Switch Gene 2 Regulates Adipose Lipolysis Through Association With Adipose Triglyceride Lipase,” Cell Metabolism 11 (2010): 194–205. 20197052 10.1016/j.cmet.2010.02.003 PMC3658843

[165] A. Sathyanarayan , M. T. Mashek , and D. G. Mashek , “ATGL Promotes Autophagy/Lipophagy via SIRT1 to Control Hepatic Lipid Droplet Catabolism,” Cell Reports 19 (2017): 1–9. 28380348 10.1016/j.celrep.2017.03.026 PMC5396179

[166] A. R. Althaher , “An Overview of Hormone‐Sensitive Lipase (HSL),” Scientific World Journal 2022 (2022): 1964684. 36530555 10.1155/2022/1964684 PMC9754850

[167] G. Fredrikson , P. Strålfors , N. O. Nilsson , and P. Belfrage , “Hormone‐Sensitive Lipase of Rat Adipose Tissue. Purification and Some Properties,” Journal of Biological Chemistry 256 (1981): 6311–6320. 7240206

[168] R. Zechner , P. C. Kienesberger , G. Haemmerle , R. Zimmermann , and A. Lass , “Adipose Triglyceride Lipase and the Lipolytic Catabolism of Cellular Fat Stores,” Journal of Lipid Research 50 (2009): 3–21. 18952573 10.1194/jlr.R800031-JLR200

[169] J. W. E. Jocken , D. Langin , E. Smit , et al., “Adipose Triglyceride Lipase and Hormone‐Sensitive Lipase Protein Expression Is Decreased in the Obese Insulin‐Resistant State,” Journal of Clinical Endocrinology & Metabolism 92 (2007): 2292–2299. 17356053 10.1210/jc.2006-1318

[170] D. Langin , A. Dicker , G. Tavernier , et al., “Adipocyte Lipases and Defect of Lipolysis in Human Obesity,” Diabetes 54 (2005): 3190–3197. 16249444 10.2337/diabetes.54.11.3190

[171] H. De Naeyer , D. M. Ouwens , Y. Van Nieuwenhove , et al., “Combined Gene and Protein Expression of Hormone‐Sensitive Lipase and Adipose Triglyceride Lipase, Mitochondrial Content, and Adipocyte Size in Subcutaneous and Visceral Adipose Tissue of Morbidly Obese Men,” Obesity Facts 4 (2011): 407–416. 22166762 10.1159/000333445 PMC6450043

[172] K. Jaworski , E. Sarkadi‐Nagy , R. E. Duncan , M. Ahmadian , and H. S. Sul , “Regulation of Triglyceride Metabolism. IV. Hormonal Regulation of Lipolysis in Adipose Tissue,” American Journal of Physiology‐Gastrointestinal and Liver Physiology 293 (2007): G1–G4. 17218471 10.1152/ajpgi.00554.2006 PMC2887286

[173] R. De Matteis , J. Arch , M. Petroni , D. Ferrari , S. Cinti , and M. Stock , “Immunohistochemical Identification of the β3‐Adrenoceptor in Intact Human Adipocytes and Ventricular Myocardium: Effect of Obesity and Treatment With Ephedrine and Caffeine,” International Journal of Obesity 26 (2002): 1442–1450. 12439645 10.1038/sj.ijo.0802148

[174] B. Buemann , S. Toubro , and A. Astrup , “Effects of the Two β3‐Agonists, ZD7114 and ZD2079 on 24 Hour Energy Expenditure and Respiratory Quotient in Obese Subjects,” International Journal of Obesity 24 (2000): 1553–1560. 11126205 10.1038/sj.ijo.0801452

[175] J. W. E. Jocken , G. H. Goossens , A. M. J. van Hees , et al., “Effect of Beta‐Adrenergic Stimulation on Whole‐Body and Abdominal Subcutaneous Adipose Tissue Lipolysis in Lean and Obese Men,” Diabetologia 51 (2008): 320–327. 18060661 10.1007/s00125-007-0866-y PMC2170457

[176] S. Kersten , “Physiological Regulation of Lipoprotein Lipase,” Biochimica et Biophysica Acta (BBA) – Molecular and Cell Biology of Lipids 1841 (2014): 919–933. 24721265 10.1016/j.bbalip.2014.03.013

[177] C. K. Roberts , R. J. Barnard , K. H. Liang , and N. D. Vaziri , “Effect of Diet on Adipose Tissue and Skeletal Muscle VLDL Receptor and LPL: Implications for Obesity and Hyperlipidemia,” Atherosclerosis 161 (2002): 133–141. 11882325 10.1016/s0021-9150(01)00622-0

[178] R. Shyam , P. Vachali , A. Gorusupudi , K. Nelson , and P. S. Bernstein , “All Three Human Scavenger Receptor Class B Proteins Can Bind and Transport All Three Macular Xanthophyll Carotenoids,” Archives of Biochemistry and Biophysics 634 (2017): 21–28. 28947101 10.1016/j.abb.2017.09.013 PMC5698089

[179] H. Shu , Y. Peng , W. Hang , J. Nie , N. Zhou , and D. W. Wang , “The Role of CD36 in Cardiovascular Disease,” Cardiovascular Research 118 (2022): 115–129. 33210138 10.1093/cvr/cvaa319 PMC8752351

[180] A. G. S. Baillie , C. T. Coburn , and N. A. Abumrad , “Reversible Binding of Long‐Chain Fatty Acids to Purified FAT, the Adipose CD36 Homolog,” Journal of Membrane Biology 153 (1996): 75–81. 8694909 10.1007/s002329900111

[181] L. P. Bechmann , R. K. Gieseler , J. P. Sowa , et al., “Apoptosis Is Associated With CD36/Fatty Acid Translocase Upregulation in Non‐Alcoholic Steatohepatitis,” Liver International 30 (2010): 850–859. 20408954 10.1111/j.1478-3231.2010.02248.x

[182] A. Bonen , N. N. Tandon , J. F. C. Glatz , J. J. F. P. Luiken , and G. J. F. Heigenhauser , “The Fatty Acid Transporter FAT/CD36 Is Upregulated in Subcutaneous and Visceral Adipose Tissues in Human Obesity and Type 2 Diabetes,” International Journal of Obesity 30, no. 2005 (2006): 877–883. 16418758 10.1038/sj.ijo.0803212

[183] R. Wu , Y. Dong , P. Yang , et al., “CD36‐ and Obesity‐Associated Granulosa Cells Dysfunction,” Reproduction, Fertility, and Development 31 (2019): 993–1001. 30832758 10.1071/RD18292

[184] X. Luo , Y. Li , P. Yang , et al., “Obesity Induces Preadipocyte CD36 Expression Promoting Inflammation via the Disruption of Lysosomal Calcium Homeostasis and Lysosome Function,” EBioMedicine 56 (2020): 102797. 32516742 10.1016/j.ebiom.2020.102797 PMC7281849

[185] H. Douglas Braymer , H. Zachary , A. L. Schreiber , and S. D. Primeaux , “Lingual CD36 and Nutritional Status Differentially Regulate Fat Preference in Obesity‐Prone and Obesity‐Resistant Rats,” Physiology & Behavior 174 (2017): 120–127. 28302572 10.1016/j.physbeh.2017.03.015 PMC5448558

[186] A. Stahl , “A Current Review of Fatty Acid Transport Proteins (SLC27),” Pflugers Archiv European Journal of Physiology 447 (2004): 722–727. 12856180 10.1007/s00424-003-1106-z

[187] H. Doege and A. Stahl , “Protein‐Mediated Fatty Acid Uptake: Novel Insights From In Vivo Models,” Physiology 21 (2006): 259–268. 16868315 10.1152/physiol.00014.2006

[188] R. Prodanović , D. Kirovski , I. Vujanac , et al., “Obesity‐Related Prepartal Insulin Resistance in Dairy Cows Is Associated With Increased Lipin 1 and Decreased FATP 1 Expression in Skeletal Muscle,” Research in Veterinary Science 150 (2022): 189–194. 35842950 10.1016/j.rvsc.2022.04.012

[189] K. Ye , L. Li , D. Zhang , et al., “Effect of Maternal Obesity on Fetal Growth and Expression of Placental Fatty Acid Transporters,” Journal of Clinical Research in Pediatric Endocrinology 9 (2017): 300–307. 28588000 10.4274/jcrpe.4510 PMC5785635

[190] N. M. Long , D. C. Rule , M. J. Zhu , P. W. Nathanielsz , and S. P. Ford , “Maternal Obesity Upregulates Fatty Acid and Glucose Transporters and Increases Expression of Enzymes Mediating Fatty Acid Biosynthesis in Fetal Adipose Tissue Depots,” Journal of Animal Science 90 (2012): 2201–2210. 22266999 10.2527/jas.2011-4343

[191] P. D. Berk , S. L. Zhou , C. L. Kiang , D. Stump , M. Bradbury , and L. M. Isola , “Uptake of Long Chain Free Fatty Acids Is Selectively Up‐Regulated in Adipocytes of Zucker Rats With Genetic Obesity and Non‐Insulin‐Dependent Diabetes Mellitus,” Journal of Biological Chemistry 272 (1997): 8830–8835. 9079720 10.1074/jbc.272.13.8830

[192] H. C. Chiu , A. Kovacs , R. M. Blanton , et al., “Transgenic Expression of Fatty Acid Transport Protein 1 in the Heart Causes Lipotoxic Cardiomyopathy,” Circulation Research 96 (2005): 225–233. 15618539 10.1161/01.RES.0000154079.20681.B9

[193] M. Marotta , A. Ferrer‐Martnez , J. Parnau , M. Turini , K. Mace , and A. M. Gomez Foix , “Fiber Type‐ and Fatty Acid Composition‐Dependent Effects of High‐Fat Diets on Rat Muscle Triacylglyceride and Fatty Acid Transporter Protein‐1 Content,” Metabolism: Clinical and Experimental 53 (2004): 1032–1036. 15281014 10.1016/j.metabol.2004.03.011

[194] I. H. McKillop , C. A. Girardi , and K. J. Thompson , “Role of Fatty Acid Binding Proteins (FABPs) in Cancer Development and Progression,” Cellular Signalling 62 (2019): 109336. 31170472 10.1016/j.cellsig.2019.06.001

[195] A. Chmurzyńska , “The Multigene Family of Fatty Acid‐Binding Proteins (FABPs): Function, Structure and Polymorphism,” Journal of Applied Genetics 47 (2006): 39–48. 16424607 10.1007/BF03194597

[196] A. E. A. Thumser and D. C. Wilton , “The Binding of Cholesterol and Bile Salts to Recombinant Rat Liver Fatty Acid‐Binding Protein,” Biochemical Journal 320, no. Pt 3 (1996): 729–733. 9003356 10.1042/bj3200729 PMC1217991

[197] Y. Zhang , E. Rao , J. Zeng , et al., “Adipose Fatty Acid Binding Protein Promotes Saturated Fatty Acid‐Induced Macrophage Cell Death Through Enhancing Ceramide Production,” Journal of Immunology 198 (2017): 798–807. 10.4049/jimmunol.1601403 PMC5225136 27920274

[198] G. S. Hotamisligil and D. A. Bernlohr , “Metabolic Functions of FABPs—Mechanisms and Therapeutic Implications,” Nature Reviews Endocrinology 11 (2015): 592–605. 10.1038/nrendo.2015.122 PMC4578711 26260145

[199] S. Liu , D. Wu , Z. Fan , et al., “FABP4 in Obesity‐Associated Carcinogenesis: Novel Insights Into Mechanisms and Therapeutic Implications,” Frontiers in Molecular Biosciences 9 (2022): 973955. 36060264 10.3389/fmolb.2022.973955 PMC9438896

[200] R. Coleman , “Enzymes of Triacylglycerol Synthesis and Their Regulation,” Progress in Lipid Research 43 (2004): 134–176. 14654091 10.1016/s0163-7827(03)00051-1

[201] A. D. Liskiewicz , L. Marczak , K. Bogus , D. Liskiewicz , M. Przybyla , and J. Lewin‐Kowalik , “Proteomic and Structural Manifestations of Cardiomyopathy in Rat Models of Obesity and Weight Loss,” Frontiers in Endocrinology 12 (2021): 568197. 33716957 10.3389/fendo.2021.568197 PMC7945951

[202] M. Yang , J. Ge , Y. L. Liu , et al., “Sortilin‐Mediated Translocation of Mitochondrial ACSL1 Impairs Adipocyte Thermogenesis and Energy Expenditure in Male Mice,” Nature Communications 15 (2024): 7746. 10.1038/s41467-024-52218-4 PMC11374900 39232011

[203] H. D. Stierwalt , S. E. Ehrlicher , M. M. Robinson , and S. A. Newsom , “Diet and Exercise Training Influence Skeletal Muscle Long‐Chain acyl‐CoA Synthetases,” Medicine & Science in Sports & Exercise 52 (2020): 569–576. 31524824 10.1249/MSS.0000000000002164 PMC7024029

[204] H. B. Kwak , T. L. Woodlief , T. D. Green , et al., “Overexpression of Long‐Chain Acyl‐CoA Synthetase 5 Increases Fatty Acid Oxidation and Free Radical Formation While Attenuating Insulin Signaling in Primary Human Skeletal Myotubes,” International Journal of Environmental Research and Public Health 16 (2019): 1157. 30935113 10.3390/ijerph16071157 PMC6480682

[205] R. A. Memon , J. Fuller , A. H. Moser , P. J. Smith , C. Grunfeld , and K. R. Feingold , “Regulation of Putative Fatty Acid Transporters and Acyl‐CoA Synthetase in Liver and Adipose Tissue in Ob/Ob Mice,” Diabetes 48 (1999): 121–127. 9892232 10.2337/diabetes.48.1.121

[206] B. D. Hegarty , G. J. Cooney , E. W. Kraegen , and S. M. Furler , “Increased Efficiency of Fatty Acid Uptake Contributes to Lipid Accumulation in Skeletal Muscle of High Fat‐Fed Insulin‐Resistant Rats,” Diabetes 51 (2002): 1477–1484. 11978645 10.2337/diabetes.51.5.1477

[207] A. S. Pereyra , K. L. McLaughlin , K. A. Buddo , and J. M. Ellis , “Medium‐Chain Fatty Acid Oxidation Is Independent of L‐Carnitine in Liver and Kidney but Not in Heart and Skeletal Muscle,” American Journal of Physiology‐Gastrointestinal and Liver Physiology 325 (2023): G287–G294. 37461880 10.1152/ajpgi.00105.2023 PMC10642992

[208] N. Turner , C. R. Bruce , S. M. Beale , et al., “Excess Lipid Availability Increases Mitochondrial Fatty Acid Oxidative Capacity in Muscle,” Diabetes 56 (2007): 2085–2092. 17519422 10.2337/db07-0093

[209] J. Y. Kim , R. C. Hickner , R. L. Cortright , G. L. Dohm , and J. A. Houmard , “Lipid Oxidation Is Reduced in Obese Human Skeletal Muscle,” American Journal of Physiology‐Endocrinology and Metabolism 279 (2000): E1039–E1044. 11052958 10.1152/ajpendo.2000.279.5.E1039

[210] H. M. A. Javaid , E. Ko , E. J. Joo , et al., “TNFα‐Induced NLRP3 Inflammasome Mediates Adipocyte Dysfunction and Activates Macrophages Through Adipocyte‐Derived Lipocalin 2,” Metabolism: Clinical and Experimental 142 (2023): 155527. 36870601 10.1016/j.metabol.2023.155527

[211] D. Jin , J. Sun , J. Huang , et al., “TNF‐α Reduces g0s2 Expression and Stimulates Lipolysis Through PPAR‐γ Inhibition in 3T3‐L1 Adipocytes,” Cytokine 69 (2014): 196–205. 24993166 10.1016/j.cyto.2014.06.005

[212] X. H. Chen , Y. P. Zhao , M. Xue , et al., “TNF‐α Induces Mitochondrial Dysfunction in 3T3‐L1 Adipocytes,” Molecular and Cellular Endocrinology 328 (2010): 63–69. 20667497 10.1016/j.mce.2010.07.005

[213] L. Al‐Khalili , K. Bouzakri , S. Glund , F. Lönnqvist , H. A. Koistinen , and A. Krook , “Signaling Specificity of Interleukin‐6 Action on Glucose and Lipid Metabolism in Skeletal Muscle,” Molecular Endocrinology 20 (2006): 3364–3375. 16945991 10.1210/me.2005-0490

[214] C. T. Coburn , F. F. Knapp, Jr. , M. Febbraio , A. L. Beets , R. L. Silverstein , and N. A. Abumrad , “Defective Uptake and Utilization of Long Chain Fatty Acids in Muscle and Adipose Tissues of CD36 Knockout Mice,” Journal of Biological Chemistry 275 (2000): 32523–32529. 10913136 10.1074/jbc.M003826200

[215] R. E. Duncan , M. Ahmadian , K. Jaworski , E. Sarkadi‐Nagy , and H. S. Sul , “Regulation of Lipolysis in Adipocytes,” Annual Review of Nutrition 27 (2007): 79–101. 10.1146/annurev.nutr.27.061406.093734 PMC2885771 17313320

[216] M. S. Westerterp‐Plantenga , M. P. G. M. Lejeune , and E. M. R. Kovacs , “Body Weight Loss and Weight Maintenance in Relation to Habitual Caffeine Intake and Green Tea Supplementation,” Obesity Research 13 (2005): 1195–1204. 16076989 10.1038/oby.2005.142

[217] L. Kang and L. E. Nagy , “Chronic Ethanol Feeding Suppresses β‐Adrenergic Receptor‐Stimulated Lipolysis in Adipocytes Isolated From Epididymal Fat,” Endocrinology 147 (2006): 4330–4338. 16794014 10.1210/en.2006-0120 PMC1764504

[218] P. Arner , “Human Fat Cell Lipolysis: Biochemistry, Regulation and Clinical Role,” Best Practice & Research Clinical Endocrinology & Metabolism 19 (2005): 471–482. 16311212 10.1016/j.beem.2005.07.004

[219] K. H. W. Lange , “Fat Metabolism in Exercise—With Special Reference to Training and Growth Hormone Administration,” Scandinavian Journal of Medicine & Science in Sports 14 (2004): 74–99. 15043630 10.1111/j.1600-0838.2004.381.x

[220] V. Patial , S. Katoch , J. Chhimwal , et al., “Catechins Prevent Obesity‐Induced Kidney Damage by Modulating PPARgamma/CD36 Pathway and Gut‐Kidney Axis in Rats,” Life Sciences 316 (2023): 121437. 36702203 10.1016/j.lfs.2023.121437

[221] M. Liu , P. Tso , and S. C. Woods , “Receptor CD36 Links a Risk‐Associated Allele to Obesity and Metabolic Disorders,” Journal of Biological Chemistry 293 (2018): 13349–13350. 30143599 10.1074/jbc.H118.004818 PMC6109921

[222] A. W. K. Tso , A. Xu , P. C. Sham , et al., “Serum Adipocyte Fatty Acid–Binding Protein as a New Biomarker Predicting the Development of Type 2 Diabetes,” Diabetes Care 30 (2007): 2667–2672. 17620449 10.2337/dc07-0413

[223] G. Tuncman , E. Erbay , X. Hom , et al., “A Genetic Variant at the Fatty Acid‐Binding Protein aP2 Locus Reduces the Risk for Hypertriglyceridemia, Type 2 Diabetes, and Cardiovascular Disease,” Proceedings of the National Academy of Sciences 103 (2006): 6970–6975. 10.1073/pnas.0602178103 PMC1447594 16641093

[224] R. Zhu and S. Chen , “Proteomic Analysis Reveals Semaglutide Impacts Lipogenic Protein Expression in Epididymal Adipose Tissue of Obese Mice,” Frontiers in Endocrinology 14 (2023): 1095432. 37025414 10.3389/fendo.2023.1095432 PMC10070826

[225] J. Nan , J. S. Lee , S. A. Lee , D. S. Lee , K. S. Park , and S. S. Chung , “An Essential Role of the N‐Terminal Region of ACSL1 in Linking Free Fatty Acids to Mitochondrial β‐Oxidation in C2C12 Myotubes,” Molecules and Cells 44 (2021): 637–646. 34511469 10.14348/molcells.2021.0077 PMC8490201

[226] M. G. Dominguez‐Bello , F. Godoy‐Vitorino , R. Knight , and M. J. Blaser , “Role of the Microbiome in Human Development,” Gut 68 (2019): 1108–1114. 30670574 10.1136/gutjnl-2018-317503 PMC6580755

[227] R. B. Sartor , “Microbial Influences in Inflammatory Bowel Diseases,” Gastroenterology 134 (2008): 577–594. 18242222 10.1053/j.gastro.2007.11.059

[228] J. Yang , H. Wei , Y. Zhou , et al., “High‐Fat Diet Promotes Colorectal Tumorigenesis Through Modulating Gut Microbiota and Metabolites,” Gastroenterology 162 (2022): 135–149.e2. 34461052 10.1053/j.gastro.2021.08.041

[229] T. R. Sampson , J. W. Debelius , T. Thron , et al., “Gut Microbiota Regulate Motor Deficits and Neuroinflammation in a Model of Parkinson's Disease,” Cell 167 (2016): 1469–1480.e12. 27912057 10.1016/j.cell.2016.11.018 PMC5718049

[230] F. Mangiola , “Gut Microbiota in Autism and Mood Disorders,” World Journal of Gastroenterology 22 (2016): 361–368. 26755882 10.3748/wjg.v22.i1.361 PMC4698498

[231] H. Weng , L. Deng , T. Wang , et al., “Humid Heat Environment Causes Anxiety‐Like Disorder via Impairing Gut Microbiota and Bile Acid Metabolism in Mice,” Nature Communications 15 (2024): 5697. 10.1038/s41467-024-49972-w PMC11228019 38972900

[232] P. J. Turnbaugh , V. K. Ridaura , J. J. Faith , F. E. Rey , R. Knight , and J. I. Gordon , “The Effect of Diet on the Human Gut Microbiome: A Metagenomic Analysis in Humanized Gnotobiotic Mice,” Science Translational Medicine 1, no. 6 (2009): 6ra14. 10.1126/scitranslmed.3000322 PMC2894525 20368178

[233] N. Qin , G. Song , X. Ren , et al., “Fish Oil Extracted From Coregonus Peled Improves Obese Phenotype and Changes Gut Microbiota in a High‐Fat Diet‐Induced Mouse Model of Recurrent Obesity,” Food & Function 11 (2020): 6158–6169. 32578655 10.1039/d0fo00911c

[234] L. A. David , C. F. Maurice , R. N. Carmody , et al., “Diet Rapidly and Reproducibly Alters the Human Gut Microbiome,” Nature 505 (2014): 559–563. 24336217 10.1038/nature12820 PMC3957428

[235] K. Parasram , A. Zuccato , M. Shin , et al., “The Emergence of Circadian Timekeeping in the Intestine,” Nature Communications 15 (2024): 1788. 10.1038/s41467-024-45942-4 PMC10899604 38413599

[236] V. Leone , S. M. Gibbons , K. Martinez , et al., “Effects of Diurnal Variation of Gut Microbes and High‐Fat Feeding on Host Circadian Clock Function and Metabolism,” Cell Host & Microbe 17 (2015): 681–689. 25891358 10.1016/j.chom.2015.03.006 PMC4433408

[237] V. K. Ridaura , J. J. Faith , F. E. Rey , et al., “Gut Microbiota From Twins Discordant for Obesity Modulate Metabolism in Mice,” Science 341 (2013): 1241214. 24009397 10.1126/science.1241214 PMC3829625

[238] P. V. Bauer , S. C. Hamr , and F. A. Duca , “Regulation of Energy Balance by a Gut‐Brain Axis and Involvement of the Gut Microbiota,” Cellular and Molecular Life Sciences 73 (2016): 737–755. 26542800 10.1007/s00018-015-2083-z PMC11108299

[239] A. Cuevas‐Sierra , O. Ramos‐Lopez , J. I. Riezu‐Boj , F. I. Milagro , and J. A. Martinez , “Diet, Gut Microbiota, and Obesity: Links With Host Genetics and Epigenetics and Potential Applications,” Advances in Nutrition 10 (2019): S17–S30. 30721960 10.1093/advances/nmy078 PMC6363528

[240] R. E. Ley , P. J. Turnbaugh , S. Klein , and J. I. Gordon , “Human Gut Microbes Associated With Obesity,” Nature 444 (2006): 1022–1023. 17183309 10.1038/4441022a

[241] F. Magne , M. Gotteland , L. Gauthier , et al., “The Firmicutes/Bacteroidetes Ratio: A Relevant Marker of Gut Dysbiosis in Obese Patients?,” Nutrients 12 (2020): 1474. 32438689 10.3390/nu12051474 PMC7285218

[242] R. Jumpertz , D. S. Le , P. J. Turnbaugh , et al., “Energy‐Balance Studies Reveal Associations Between Gut Microbes, Caloric Load, and Nutrient Absorption in Humans,” American Journal of Clinical Nutrition 94 (2011): 58–65. 21543530 10.3945/ajcn.110.010132 PMC3127503

[243] P. J. Turnbaugh , M. Hamady , T. Yatsunenko , et al., “A Core Gut Microbiome in Obese and Lean Twins,” Nature 457 (2009): 480–484. 19043404 10.1038/nature07540 PMC2677729

[244] J. Hu , P. Guo , R. Mao , et al., “Gut Microbiota Signature of Obese Adults Across Different Classifications,” Diabetes, Metabolic Syndrome and Obesity: Targets and Therapy 15 (2022): 3933–3947. 36601354 10.2147/DMSO.S387523 PMC9807070

[245] N. Fei and L. Zhao , “An Opportunistic Pathogen Isolated From the Gut of an Obese Human Causes Obesity in Germfree Mice,” ISME Journal 7, no. 4 (2013): 880–884. 23235292 10.1038/ismej.2012.153 PMC3603399

[246] D. P. Patil , D. P. Dhotre , S. G. Chavan , et al., “Molecular Analysis of Gut Microbiota in Obesity Among Indian Individuals,” Journal of Biosciences 37 (2012): 647–657. 22922190 10.1007/s12038-012-9244-0

[247] A. Ignacio , M. R. Fernandes , V. A. A. Rodrigues , et al., “Correlation Between Body Mass Index and Faecal Microbiota From Children,” Clinical Microbiology and Infection 22 (2016): 258.e1–258.e8. 10.1016/j.cmi.2015.10.031 26551842

[248] M. Befus , F. D. Lowy , B. A. Miko , D. V. Mukherjee , C. T. A. Herzig , and E. L. Larson , “Obesity as a Determinant of *Staphylococcus aureus* Colonization Among Inmates in Maximum‐Security Prisons in New York State,” American Journal of Epidemiology 182 (2015): 494–502. 26292691 10.1093/aje/kwv062 PMC4564937

[249] A. Santacruz , M. C. Collado , L. García‐Valdés , et al., “Gut Microbiota Composition Is Associated With Body Weight, Weight Gain and Biochemical Parameters in Pregnant Women,” British Journal of Nutrition 104 (2010): 83–92. 20205964 10.1017/S0007114510000176

[250] X. Niu , Q. Zhang , J. Liu , et al., “Effect of Synbiotic Supplementation on Obesity and Gut Microbiota in Obese Adults: A Double‐Blind Randomized Controlled Trial,” Frontiers in Nutrition 11 (2024): 1510318. 39664910 10.3389/fnut.2024.1510318 PMC11633458

[251] Y. Gu , C. Liu , N. Zheng , W. Jia , W. Zhang , and H. Li , “Metabolic and Gut Microbial Characterization of Obesity‐Prone Mice Under a High‐Fat Diet,” Journal of Proteome Research 18 (2019): 1703–1714. 30793608 10.1021/acs.jproteome.8b00945

[252] Y. Wan , F. Wang , J. Yuan , et al., “Effects of Dietary Fat on Gut Microbiota and Faecal Metabolites, and Their Relationship With Cardiometabolic Risk Factors: A 6‐Month Randomised Controlled‐Feeding Trial,” Gut 68 (2019): 1417–1429. 30782617 10.1136/gutjnl-2018-317609

[253] J. M. Monk , W. Wu , D. Lepp , et al., “Navy Bean Supplemented High‐Fat Diet Improves Intestinal Health, Epithelial Barrier Integrity and Critical Aspects of the Obese Inflammatory Phenotype,” Journal of Nutritional Biochemistry 70 (2019): 91–104. 31195365 10.1016/j.jnutbio.2019.04.009

[254] M. A. Mushref and S. Srinivasan , “Effect of High Fat‐Diet and Obesity on Gastrointestinal Motility,” Annals of Translational Medicine 1 (2013): 14. 24432301 10.3978/j.issn.2305-5839.2012.11.01 PMC3890396

[255] R. Nagpal , T. M. Newman , S. Wang , S. Jain , J. F. Lovato , and H. Yadav , “Obesity‐Linked Gut Microbiome Dysbiosis Associated With Derangements in Gut Permeability and Intestinal Cellular Homeostasis Independent of Diet,” Journal of Diabetes Research 2018 (2018): 3462092. 30250849 10.1155/2018/3462092 PMC6140100

[256] W. A. Walters , Z. Xu , and R. Knight , “Meta‐Analyses of Human Gut Microbes Associated With Obesity and IBD,” FEBS Letters 588 (2014): 4223–4233. 25307765 10.1016/j.febslet.2014.09.039 PMC5050012

[257] I. Liaqat , S. Naseem , S. Eijaz , et al., “Impact of Interplay Between Obese Gut Microbiota and Diet in Developing Obesity in Synthetic Community Mice,” Journal of Oleo Science 70 (2021): 1285–1293. 34483221 10.5650/jos.ess21148

[258] J. M. S. Lemons and L. Liu , “Chewing the Fat With Microbes: Lipid Crosstalk in the Gut,” Nutrients 14 (2022): 573. 35276931 10.3390/nu14030573 PMC8840455

[259] S. P. Mishra , B. Wang , S. Jain , et al., “A Mechanism by Which Gut Microbiota Elevates Permeability and Inflammation in Obese/Diabetic Mice and Human Gut,” Gut 72 (2023): 1848–1865. 36948576 10.1136/gutjnl-2022-327365 PMC10512000

[260] F. Salas‐Perez , T. Assmann , O. Ramos‐Lopez , J. Martínez , J. Riezu‐Boj , and F. Milagro , “Crosstalk Between Gut Microbiota and Epigenetic Markers in Obesity Development: Relationship Between Ruminococcus, BMI, and MACROD2/SEL1L2 Methylation,” Nutrients 15 (2023): 1550. 37049393 10.3390/nu15071550 PMC10097304

[261] B. Chassaing , R. E. Ley , and A. T. Gewirtz , “Intestinal Epithelial Cell Toll‐Like Receptor 5 Regulates the Intestinal Microbiota to Prevent Low‐Grade Inflammation and Metabolic Syndrome in Mice,” Gastroenterology 147 (2014): 1363–1377.e17. 25172014 10.1053/j.gastro.2014.08.033 PMC4253564

[262] I. Rurik , P. Apor , M. Barna , et al., “Az elhízás kezelése és megelőzése: táplálkozás, testmozgás, orvosi lehetőségek: Hazai szakmaközi ajánlás,” Orvosi Hetilap 162 (2021): 323–335. 33640874 10.1556/650.2021.32020

[263] S. Paccosi , B. Cresci , L. Pala , C. M. Rotella , and A. Parenti , “Obesity Therapy: How and Why?,” Current Medicinal Chemistry 27 (2020): 174–186. 30678612 10.2174/0929867326666190124121725

[264] J. Miyamoto , M. Igarashi , K. Watanabe , et al., “Gut Microbiota Confers Host Resistance to Obesity by Metabolizing Dietary Polyunsaturated Fatty Acids,” Nature Communications 10 (2019): 4007. 10.1038/s41467-019-11978-0 PMC6728375 31488836

[265] H. Zhang , Y. Xie , F. Cao , and X. Song , “Gut Microbiota‐Derived Fatty Acid and Sterol Metabolites: Biotransformation and Immunomodulatory Functions,” Gut Microbes 16 (2024): 2382336. 39046079 10.1080/19490976.2024.2382336 PMC11271093

[266] H. Shiratori , H. Oguchi , Y. Isobe , et al., “Gut Microbiota‐Derived Lipid Metabolites Facilitate Regulatory T Cell Differentiation,” Scientific Reports 13 (2023): 8903. 37264064 10.1038/s41598-023-35097-5 PMC10235104

[267] M. Longo , F. Zatterale , J. Naderi , et al., “Adipose Tissue Dysfunction as Determinant of Obesity‐Associated Metabolic Complications,” International Journal of Molecular Sciences 20 (2019): 2358. 31085992 10.3390/ijms20092358 PMC6539070

[268] S. G. Shitole , M. L. Biggs , J. H. Ix , et al., “Fasting and Postload Nonesterified Fatty Acids and Glucose Dysregulation in Older Adults,” American Journal of Epidemiology 191 (2022): 1235–1247. 35247051 10.1093/aje/kwac044 PMC9989335

[269] T. Takeuchi , K. Kameyama , E. Miyauchi , et al., “Fatty Acid Overproduction by Gut Commensal Microbiota Exacerbates Obesity,” Cell Metabolism 35 (2023): 361–375.e9. 36652945 10.1016/j.cmet.2022.12.013

[270] C. Rosso , K. Kazankov , R. Younes , et al., “Crosstalk Between Adipose Tissue Insulin Resistance and Liver Macrophages in Non‐Alcoholic Fatty Liver Disease,” Journal of Hepatology 71 (2019): 1012–1021. 31301321 10.1016/j.jhep.2019.06.031

[271] N. K. Newman , Y. Zhang , J. Padiadpu , et al., “Reducing Gut Microbiome‐Driven Adipose Tissue Inflammation Alleviates Metabolic Syndrome,” Microbiome 11 (2023): 208. 37735685 10.1186/s40168-023-01637-4 PMC10512512

[272] A. W. C. Man , Y. Zhou , N. Xia , and H. Li , “Involvement of Gut Microbiota, Microbial Metabolites and Interaction With Polyphenol in Host Immunometabolism,” Nutrients 12 (2020): 3054. 33036205 10.3390/nu12103054 PMC7601750

